# Efficacy and safety of neoadjuvant immunotherapy plus chemotherapy followed by adjuvant immunotherapy in resectable non-small cell lung cancer: a meta-analysis of phase 3 clinical trials

**DOI:** 10.3389/fimmu.2024.1359302

**Published:** 2024-04-05

**Authors:** Wenjing Zhang, Zhanpeng Liang, Yurong Zhao, Yanwei Li, Ting Chen, Wenxia Li, Yunqi Chen, Peiye Wu, Huatang Zhang, Cantu Fang, Luzhen Li

**Affiliations:** Department of Oncology, Zhongshan Hospital of Traditional Chinese Medicine Affiliated to Guangzhou University of Traditional Chinese Medicine, Guangdong, China

**Keywords:** perioperative immunotherapy, immune checkpoint inhibitors, chemotherapy, resectable non-small cell lung cancer, meta-analysis

## Abstract

**Objective:**

At present, several important trials have been published show that perioperative immunotherapy combined with chemotherapy can improve the prognosis of patients with resectable non-small cell lung cancer, which further optimizes treatment options. Therefore, we conducted a systematic review and meta-analysis to evaluate the efficacy and safety of perioperative immunotherapy combined with chemotherapy in resectable non-small cell lung cancer.

**Methods:**

The following databases were searched for relevant studies: PubMed, EMBASE, Cochrane library (updated 12 October 2023). All randomized trials comparing perioperative immunotherapy combined with chemotherapy versus chemotherapy alone in resectable non-small cell lung cancer were eligible for inclusion. Data were analyzed using Review Manager 5.4.1 (Cochrane collaboration software). Primary outcomes and measures included overall survival (OS), event-free survival (EFS), pathological complete response (pCR), major pathological response (MPR), R0 resection rate, rate of underwent surgery and adverse events (AEs).

**Results:**

A total of 2912 patients (1453 receiving perioperative immunotherapy plus chemotherapy and 1459 receiving chemotherapy alone) were included in this systematic review and meta-analysis. The result showed that compared with chemotherapy alone, combined therapy significantly improved OS (HR = 0.68;95% CI: 0.56-0.83), EFS (HR = 0.58;95% CI: 0.51-0.65), pCR (OR = 7.53;95% CI: 4.63-12.26), MPR (OR = 5.03;95% CI: 3.40-7.44), R0 resection (OR = 1.58;95% CI: 1.152.18) and rate of underwent surgery (OR = 1.25;95% CI: 1.04-1.49). However, combination therapy was associated with higher risk of severe adverse event (OR = 1.46;95% CI: 1.19-1.78; P=0.0002), grade 3 and higher treatment-related adverse event (TRAE) (OR = 1.25;95% CI: 1.06-1.49; P=0.010), TRAE that led to interruption (OR = 1.90;95% CI: 1.34-2.68; P=0.0003) and immune-related adverse event (OR = 2.78;95% CI: 2.18-3.55; P<0.00001). Significant benefits were observed across most subgroups of EFS and pCR. However, no statistical differences were observed for EFS of never smoked (HR = 0.73;95% CI: 0.51-1.05) and EGFR-mutation positive (HR = 0.35;95% CI: 0.04-3.03).

**Conclusion:**

This systematic review and meta-analysis found superior efficacy associated with perioperative immunotherapy plus chemotherapy compared with chemotherapy alone in both tumor regression and prolonged survival in resectable NSCLC, but increased the risk of TRAE, so monitoring for adverse events is warranted.

**Systematic review registration:**

https://www.crd.york.ac.uk/prospero, identifier (CRD42023476786).

## 1 Introduction

Lung cancer is one of the most common malignancies in the world and one of the primary causes of death, among which non-small cell lung cancer (NSCLC) accounts for approximately 85% of all lung cancer diagnoses (1, 2). About 20% of patients with NSCLC are diagnosed at stage I or II, which are eligible for surgical resection (3). However, 80% of the patients with advanced NSCLC diagnosed at stage III or IV, meaning that they are not suitable for surgical resection (4). For resectable NSCLC, surgery is still the most common treatment option (5). Nonetheless, for unresectable NSCLC, the survival benefit of surgery is not ideal. In addition, the occurrence of local recurrence early after surgery poses great challenges to the long-term survival of patients. Resectable NSCLC is a refractory disease with a poor prognosis and a 5-year survival rate of just 36% (6). Currently, the progress of radiotherapy, chemotherapy, immunization and targeted therapy has improved the survival of patients with resectable NSCLC (7). However, results from an important phase III randomized trial showed that neoadjuvant chemoradiotherapy associated with superior OS, pCR, and R0 resection compared with chemotherapy alone. Nevertheless, neoadjuvant chemoradiotherapy did not result in longer EFS and OS, but pCR was still as high as 16% (8, 9). In addition, targeted therapy decreased the risk of postoperative recurrence, and the resection rate was higher than that traditional neoadjuvant chemotherapy containing platinum, but pCR had not been observed (10, 11). Therefore, how to optimize the treatment strategy has become a crucial topic to explore urgently. In recent years, neoadjuvant immunotherapy has increasingly become the focus of treatment for resectable NSCLC. Compared with chemoradiotherapy and targeted therapy, neoadjuvant immunotherapy can not only significantly reduce tumor size, but also bring greater survival benefit to patients (12). Previous studies have demonstrated the potential benefits of immunotherapy at different stages of NSCLC. For patients with high expression of PDL-1, PD-1 inhibitors significantly prolonged the median OS in first-line treatment, which showed better benefits than chemotherapy (13, 14). In second-line treatment, immunotherapy also demonstrated a significant survival benefit (15). Especially in recent years, abundant evidence-based medical evidence has been accumulated in many Exploratory research, such as neoadjuvant immunotherapy and chemotherapy, double-adjuvant immunotherapy and immune monotherapy. CheckMate-816 (16) was the first phase III clinical trial to evaluate the safety and efficacy of neoadjuvant immunotherapy combined with chemotherapy versus chemotherapy alone in resectable NSCLC. This study utilized neoadjuvant nivolumab in combination with chemotherapy without postoperative adjuvant immunotherapy. Analysis of OS showed that neoadjuvant immunotherapy plus chemotherapy decreased the risk of death and distant metastasis. Besides, there is a trend of OS benefits. Another study, IMpower010 (17), confirmed that adjuvant immunotherapy that perioperative immunotherapy significantly improved the pCR and OS in resectable NSCLC. However, the potential beneficiary population for perioperative immunotherapy plus chemotherapy is currently not well defined, and the safety and efficacy of this treatment still need to be evaluated by brought survival benefits to NSCLC which indicated that the combination of neoadjuvant immunotherapy and adjuvant immunotherapy is potentially beneficial. Based on CheckMate-816 and IMpower010, the combination of neoadjuvant immunotherapy and adjuvant immunotherapy may be a promising therapeutic method. NADIM II (18), a phase II clinical study, confirmed the sandwich cake scheme of neoadjuvant immunotherapy combined with chemotherapy followed by adjuvant therapy achieved a full range of therapeutic benefits in pCR, PFS and OS. This provides preliminary evidence for the potential value of neoadjuvant immunotherapy in the treatment of NSCLC. However, phase II clinical data are not yet mature. So more randomized controlled phase III trials are needed to ensure the efficacy of this treatment strategy (19). In addition, while neoadjuvant immunotherapy combined with chemotherapy has some advantages in patients with resectable small cell lung cancer, its safety still remains some uncertainty and requires further exploration (20). Based on this, we conducted a systematic review and meta-analysis to evaluate the efficacy and safety of perioperative immunotherapy plus chemotherapy versus chemotherapy alone in resectable NSCLC.

## 2 Methods

This study was registered in the PROSPERO database (CRD42023476786) and was conducted according to the preferred reporting project for systematic review and meta-analysis (PRISMA) statement (21). And this study aims to compare the efficacy and safety of perioperative immunotherapy plus chemotherapy with chemotherapy alone in resectable NSCLC.

The PICOS criteria of this meta-analysis are as follows:

Participants: patients with cytological or pathologic diagnoses of resectable non-small cell lung cancer (NSCLC).
Intervention: neoadjuvant immunotherapy combined with chemotherapy followed by adjuvant immunotherapy.
Control: neoadjuvant chemotherapy and placebo followed by placebo.
Outcomes: event-free survival (EFS) and overall survival (OS), which were reported in the form of hazard ratios. In addition, pathological complete response rate (PCR), major pathological response rate (MPR), R0 resection rate, and adverse events (AEs).
Study design: randomized controlled Phase III trial.

### 2.1 Search strategy

A comprehensive search of records through the PubMed, Embase and Cochrane Library databases was carried out (date of the last search: October 12, 2023). The keywords or corresponding grid terms used to search the database are: perioperative, immune checkpoint inhibitors, chemotherapy, resectable non-small cell lung cancer. The relevant bibliography of candidate articles was manually searched to identify additional studies. The proceedings of the American Society of Clinical Oncology (ASCO) and the European Society of Medical Oncology (ESMO)/European Cancer Congress (ECC) annual meetings were searched for abstract reports of relevant studies. If there was any overlapping data, the most complete and updated report was selected for inclusion in this meta-analysis. Additionally, the references from all eligible studies were manually reviewed to identify any other relevant studies.

### 2.2 Eligibility criteria

The inclusion criteria used to select studies in this meta-analysis were (1): patients with cytologic or pathological diagnosis of resectable NSCLC, (2) patients with an average age greater than 18 years, (3) Phase III prospective, randomized trials (RCTs) comparing perioperative immunotherapy plus chemotherapy with chemotherapy alone, (4) Studies reporting at least one of the following outcomes: overall survival (OS), pathological complete response (pCR), major pathological response (MPR), event-free survival (EFS), R0 resection rate, rate of underwent surgery and adverse events (AEs).

The exclusion criteria were listed below: (1) patients with inoperable non-small cell lung cancer; (2) phase II randomized trials, non-randomized controlled studies, basic research, retrospective studies, case reports, duplicate publications and studies for which no relevant data could be extracted; and (3) RCTs that were based on overlapping patients.

### 2.3 Study selection and data extraction

Two experienced investigators independently screened the records based on the established inclusion and exclusion criteria. Differences were resolved by consulting a third investigator. The investigators reviewed the literature by browsing titles and abstracts to complete an initial selection and following a full review of potentially eligible articles and the selection of eligible articles based on pre-established criteria.

Extracted data included baseline characteristics, sample size and interventions used, number of assessable patients. The primary and secondary outcome endpoints are OS, EFS, pCR, MPR, R0 resection rate, rate of underwent surgery and AEs. Two investigators independently extracted relevant data and resolved any differences by consulting a third investigator. When multiple articles contained overlapping patient series, we prioritized the extraction of outcome data from the primary articles with the largest sample size for early outcomes and the articles with the longest follow-up duration for the late outcomes.

### 2.4 Outcome

The results of this review include OS, EFS, pCR, MPR, R0 resection rate, rate of underwent surgery and AEs. OS is defined as the time from randomization to death and pCR is defined as the absence of residual tumor cells after evaluation of removed tumor tissue and regional lymph, which was often used as an alternative marker for clinical trials of neoadjuvant therapy. MPR is defined as residual tumor cells which is less than or equal to 10% by pathological examination of postoperative specimens. However, the determination of MPR is susceptible to subjective factors. EFS is defined as the time from randomization to the occurrence of any event, including disease progression, discontinuation of treatment for any reason or death. R0 resection is defined as the successful removal of the tumor during surgery and the absence of residual cancer cells at the excision margin. Underwenting surgery is defined as patients receiving surgical treatment during the course of the trial. AEs, graded according to National Cancer Institute Common Terminology Criteria for Adverse Events version 4.03.

### 2.5 Risk of bias

Two investigators independently assessed the quality of the included trials using the Cochrane Collaboration tools with respect to randomized sequence generation, assignment concealment, blinding, incomplete outcome data, and selective outcome reporting (22). Any differences in quality assessment were resolved by consulting a third investigator.

### 2.6 Statistical analysis

Data were analyzed using Review Manager 5.4.1 (Cochrane Collaboration Software). These measures were either extracted directly from the articles or calculated. PCR, MPR, R0 resection rate, rate of underwent surgery and AEs were reported as odds ratios (OR) with corresponding 95% confidence intervals (95% CI). EFS and OS were reported as hazard ratio (HR) and had 95% CI. p < 0.05 was considered statistically significant. For effectiveness or side effects, HR > 1 or OR < 1 favored chemotherapy alone (control), while HR < 1 or OR > 1 favored combination therapy (experimental). The χ² (Cochran Q) and I² statistics will be used to assess heterogeneity between studies. A fixed-effects model is used for data synthesis unless heterogeneity is large (I²>50%), in which case a random-effects model is used (23, 24). Funnel plots and an Egger test were adopted to investigate the potential for publication bias (25). Subgroup analysis was conducted for age, sex, smoking history, physical status, disease stage, pathological type, tumor cell PD-L1 expression level and region to assess the effect of combination therapy in resectable NSCLC.

## 3 Results

### 3.1 Study identification and quality assessment

**Figure 1:**
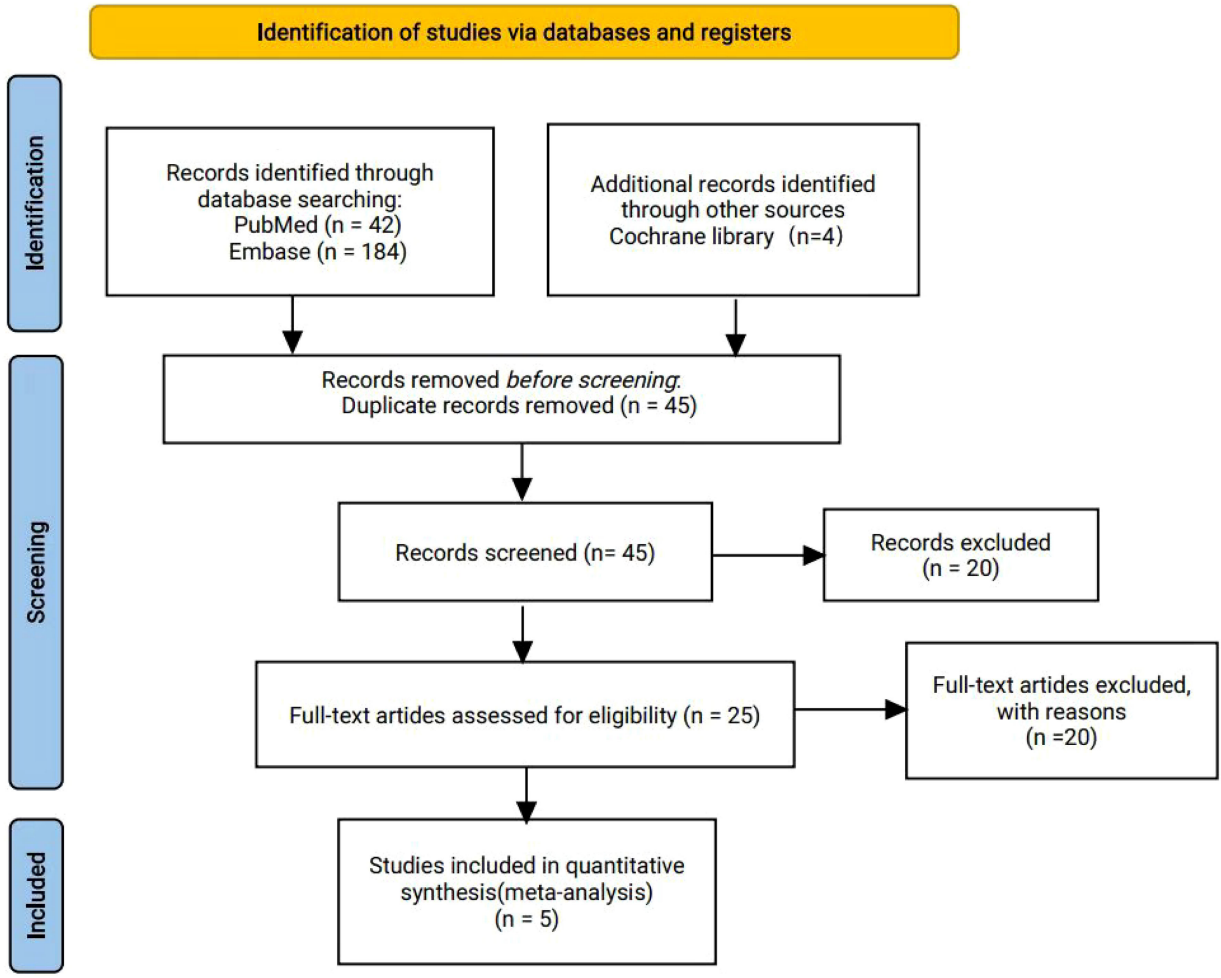
PRISMA flow diagram.

**Figure 2:**
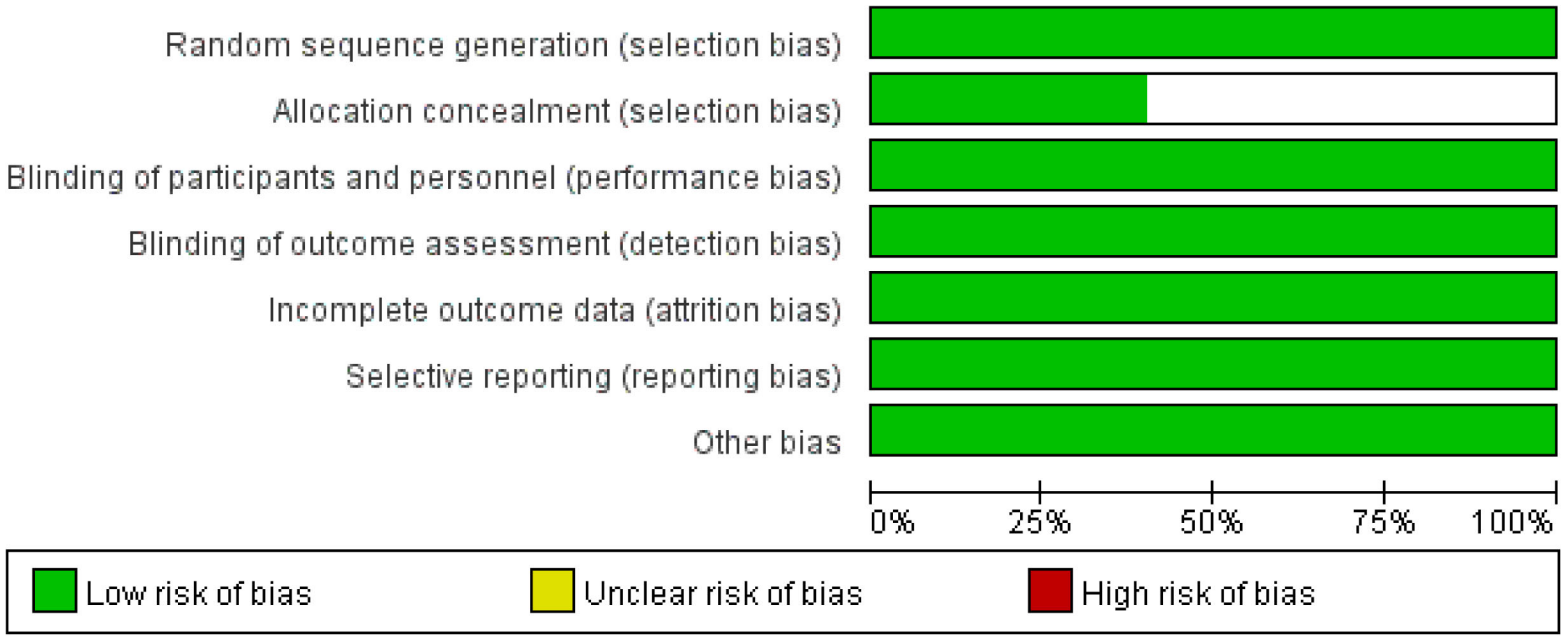
Risk of bias graph.

**Figure 3:**
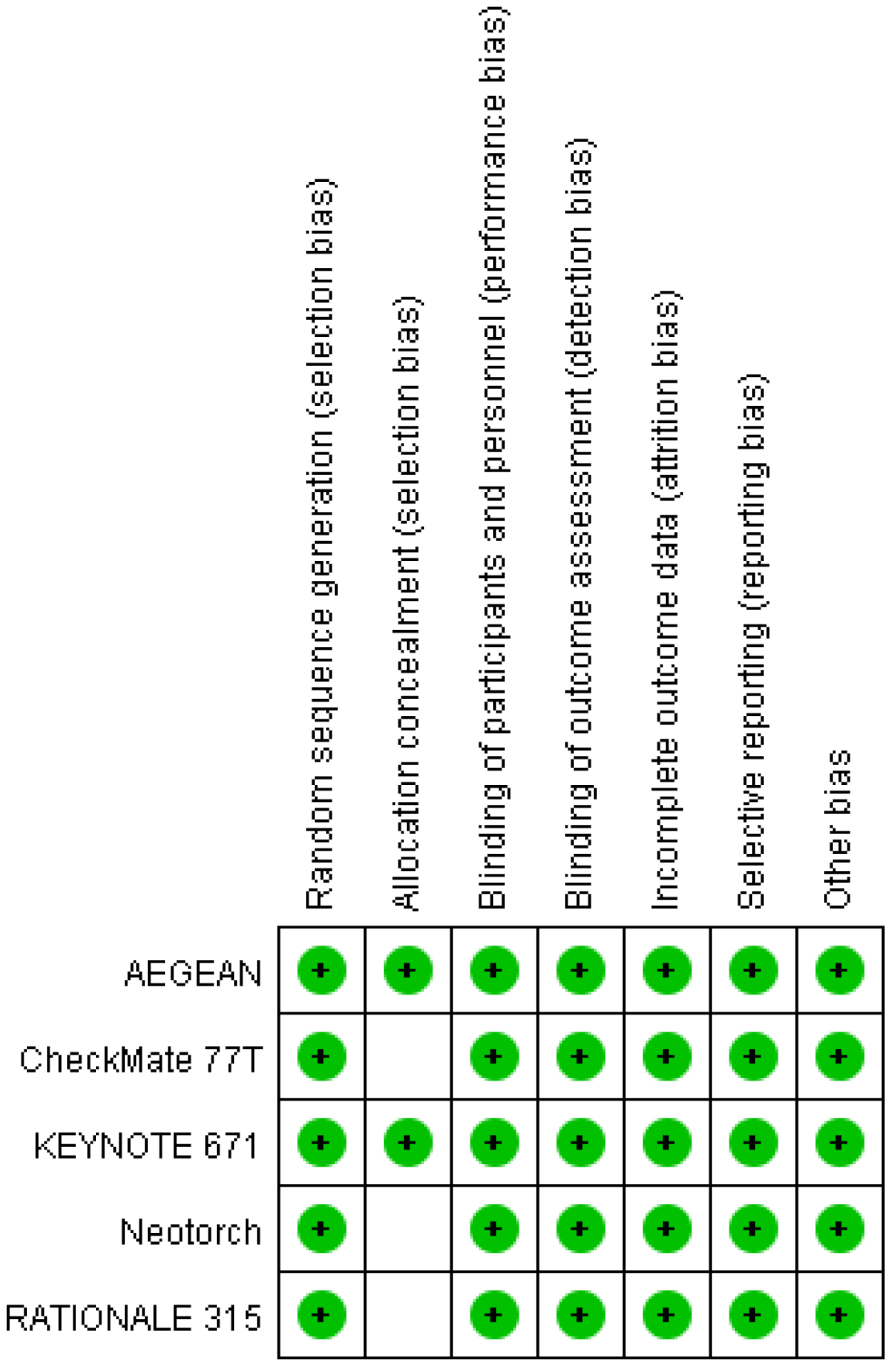
Risk of bias summary.

A total of 230 articles were retrieved from the electronic databases: PubMed, EMBASE, the Cochrane Library. After excluding 32 duplicate articles, 194 articles were initially excluded based on the review of titles and abstracts. Full texts of 25 articles were reviewed, resulting in the inclusion of 5 articles in the final analysis. This meta-analysis comprised five randomized controlled trials (26–30), involving 2855 patients. Among these, one study was fully published (26), while four trials were published only in abstract form (27–30). The PRISMA flowchart illustrating the process of study identification and selection is provided in **Figure 1**. Since all studies included were randomized, selection and loss bias were minimized (**Figures 2**, **3**).

### 3.2 Study and patient characteristics

All five studies reported detailed data on pCR and MPR. Three trials provided detailed data on OS. Five studies reported EFS. The characteristics of the five trials are reported in **Table 1**.

**Table 1:** Characteristics of included studies.

	AEGEAN	CheckMate-77T	KEYNOTE-671	NEOTORCH	RATIONALE-315
Study design	randomized controlled Phase III trial				
Enrollment stage	TNM 8th II A-IIIB[N2]	TNM 8th II A-IIIB[N2]	TNM 8th II-IIIB[N2]	TNM 8th III	TNM 8th II-III A
Number of participants	802	461	795	404	453
Preoperative schedule	D 1500 mg IV +platinum-based CT Q3W for 4 cycles VS Placebo IV +platinum-based CT Q3W for 4 cycles	N 360mg Q3W + platinum-based CT Q3W for 4 cycles VS PBO Q3W +platinum-based CT Q3W for 4 cycles	P 200 mg IV Q3W + platinum-based CT for 4 cycles VS PBO Q3W+ GP or PP for 4 cycles	Tor 240mg IV + platinum-based CT Q3W for 3 cycles VS PBO + platinum-based CT Q3W for 3 cycles	Tis 200 mg IV Q3W + platinum-based CT Q3W for 3-4 cycles + VS PBO + platinum-based CT Q3W for 3-4 cycles
Postoperative schedule	D 1500 mg IV Q4W for 12 cycles VS PBO Q4W for 12 cycles	N 480mg Q4W for 1 year VS PBO Q4W for 1 year	P 200 mg IV Q3W for 13 cycles VS PBO Q3W for 13 cycles	Tor 240mg IV + platinum-based CT Q3W for 1 cycle followed by Tor 240mg IV Q3W for 13 cycles VS PBO + platinum-based CT Q3W for 1 followed by cycle PBO Q3W for 13 cycles	Tis 400mg IV Q6W for 8 cycles VS PBO IV Q6W
Primary endpoint	pCR, EFS	EFS	EFS, OS	EFS, MPR	EFS, MPR/pCR
Complete radical surgery	78% vs 77%	78% vs 77%	82% vs 79%	82% vs 73%	84% vs 76%
R0 resection	94.7% vs 91.3%	89% vs 90%	92% vs 84%	96% vs 93%	/
pCR	17.2% vs 4.3%	25.3% vs 4.7%	18.1% vs 4.0%	24.8% vs 1.0%	40.7% vs 5.7%
MPR	33.3% vs 12.3%	35.4% vs 12.1%	30.2% vs 11.0%	48.5% vs 8.4%	56.2% vs 15.0%
EFS	NR vs 25.9 m (HR=0.68)	NR vs 18.4 m (HR=0.58)	47.2 vs 18.3 m (HR=0.59)	NR vs 15.1 m (HR=0.40)	NR vs NR (HR=0.56)
grade 3 and higher AEs	42.3 vs 43.4%	32 vs 25%	45.2 vs 37.8%	63.4 vs 54.4%	69.5 vs 65.5%

pCR, pathological complete response; MPR, major pathological response (tumors with no more than 10% viable tumor cells); EFS, event-free survival; OS, overall survival; NR, not reach; CT, Computed Tomography; D, durvalumab; N, nivolumab; P, pembrolizumab; Tor, Toripalimab; Tis, Tislelizumab; NA, not available.

All five trials evaluated the prognostic effect of perioperative immunotherapy combined with chemotherapy versus chemotherapy alone in resectable non-small cell lung cancer. However, the five trials differed in the characteristics of patients included, immunosuppressant selection, dosing patterns and primary endpoints.

AEGEAN (26) enrolled 802 patients with IIA-IIIB (N2) NSCLC and no EGFR and ALK positive. The subjects were randomly divided into two groups and respectively received neoadjuvant durvalumab or placebo combined with platinum-containing chemotherapy for 4 cycles before surgery. And postoperative patients were treated with 12 cycles of durvalumab or placebo. The primary endpoints of the study were pCR and EFS.

CheckMate-77T (27) enrolled 461 patients with IIA to IIIB NSCLC and no EGFR or ALK mutations. Participants were randomly allocated into two groups that one received 4 cycles of neoadjuvant nivolumab combined with chemotherapy and the other received neoadjuvant chemotherapy plus placebo. Postoperatively, they were assigned to either 1 year of adjuvant nivolumab treatment or adjuvant placebo treatment. The primary endpoint of this study was EFS.

KEYNOTE-671 (28) enrolled 795 patients with resectable II, IIIA, and IIIB (N2) NSCLC. Participants were randomized to receive either 4 cycles of pembrolizumab combined with chemotherapy as neoadjuvant therapy or chemotherapy combined with placebo as neoadjuvant therapy. Adjuvant immunotherapy or placebo-assisted therapy were given 13 weeks after surgery. The main endpoints of this study were EFS and OS.

Neotorch (29) enrolled 404 patients with stage III NSCLC. One group received preoperative treatment consisting of 3 cycles of toripalimab combined with chemotherapy as neoadjuvant therapy, while the other group received paclitaxel combined with carboplatin chemotherapy. After surgery, 1 cycle of adjuvant treatment with toripalimab plus chemotherapy and 13 cycles of toripalimab consolidation therapy were continued. The primary endpoints of this study include EFS in stage III patients and MPR in both stage III and II-III patients.

RATIONALE-315 (30) enrolled 453 patients with resectable II-IIIA NSCLC were included and randomly divided into two groups to receive preoperative 3-4 cycles of Tislelizumab combined with platinum double-drug chemotherapy neoadjuvant immunotherapy or platinum double-drug chemotherapy. Two to eight cycles of Tislelizumab immunoadjuvant therapy or platinum-containing chemotherapy were continued after surgery. The primary endpoints of the study were EFS and MPR.

### 3.3 The primary outcome

#### 3.3.1 Overall survival

**Figure 4:**
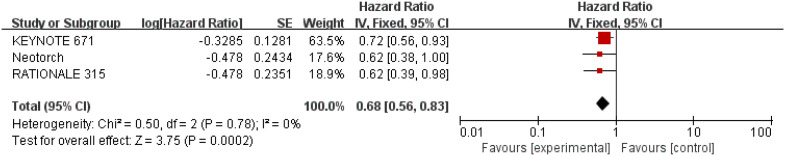
Assessment of overall survival. The diamond indicates best estimate of the true (pooled) outcome (with width indicating 95% CI); HR, hazard ratio; experimental stands for perioperative immunotherapy combined with chemotherapy; control stands for chemotherapy alone. Since there is no heterogeneity, a fixed-effects model is used.

Results for OS came from three studies (28–30) involving a total of 1,652 patients. The results showed that perioperative immunotherapy plus chemotherapy further increased OS and reduced the risk of death by 32% (HR = 0.68; 95% CI: 0.56-0.83; P = 0.0002), with no heterogeneity (Chi^2 ^= 0.50; df = 2 [p = 0.78]; I^2 ^= 0%) (**Figure 4**).

#### 3.3.2 Event-free survival

**Figure 5:**
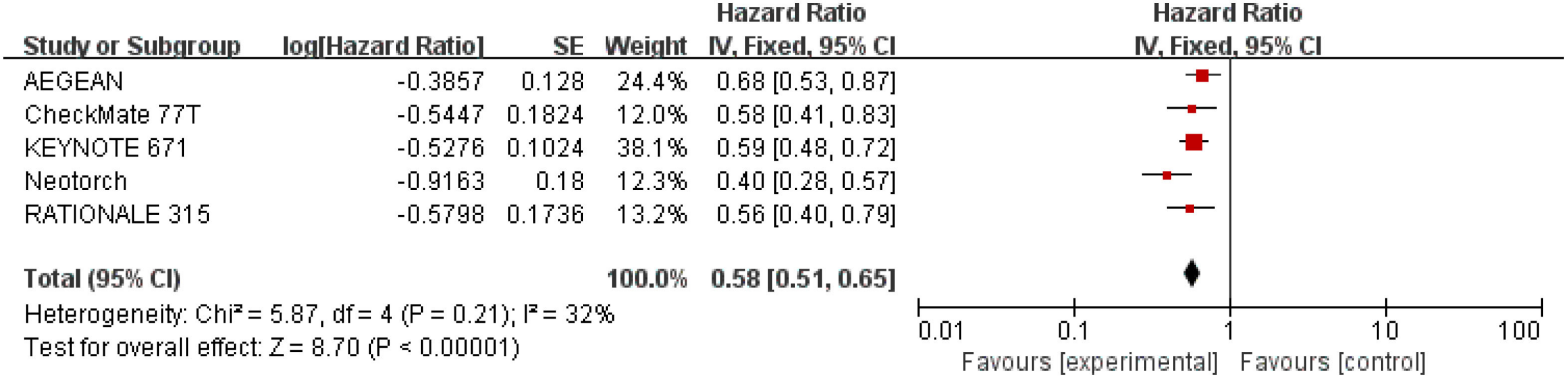
Assessment of event-free survival. The diamond indicates best estimate of the true (pooled) outcome (with width indicating 95% CI); HR, hazard ratio; experimental stands for perioperative immunotherapy combined with chemotherapy; control stands for chemotherapy alone. Since there is moderate heterogeneity, a fixed-effects model is used.

Results for EFS came from five studies (26–30) involving a total of 2,855 patients. Overall, patients receiving perioperative immunotherapy plus chemotherapy resulted in higher EFS (HR = 0.58; 95% CI: 0.51-0.65; P <0.00001). Additionally, moderate heterogeneity was found among the trials (Chi^2 ^= 5.87; df = 4 [p = 0.21]; I^2 ^= 32%) (**Figure 5**).

#### 3.3.3 Pathological complete response

**Figure 6:**
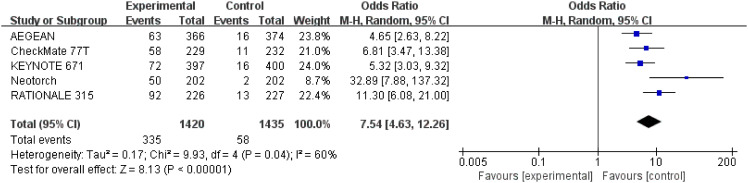
Assessment of pathological complete response. The diamond indicates best estimate of the true (pooled) outcome (with width indicating 95% CI); OR, odds ratio; experimental stands for perioperative immunotherapy combined with chemotherapy; control stands for chemotherapy alone. Since there is high heterogeneity, a random-effects model is used.

Results for pCR were extracted from 2,855 patients in three studies (28–30). The results showed that perioperative immunotherapy plus chemotherapy compared with chemotherapy alone was associated with higher pCR ((OR = 7.54;95% CI: 4.63-12.26; P <0.00001), with moderate heterogeneity (Chi^2 ^= 9.93; df = 4 [p= 0.04]; I^2 ^= 60%) (**Figure 6**).

#### 3.3.4 Major pathological response

**Figure 7:**
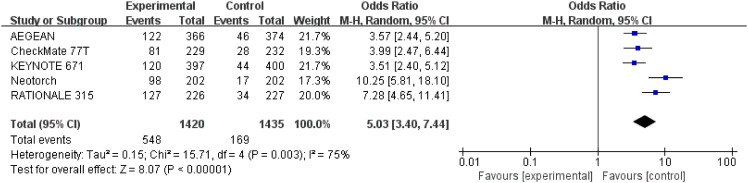
Assessment of major pathological response. The diamond indicates best estimate of the true (pooled) outcome (with width indicating 95% CI); OR, odds ratio; experimental stands for perioperative immunotherapy combined with chemotherapy; control stands for chemotherapy alone. Since there is high heterogeneity, a random-effects model is used.

Detailed data of MPR were extracted from five studies (26–30) involving a total of 2,855 patients. Perioperative immunotherapy plus chemotherapy was associated with higher MPR (OR = 5.03; 95% CI: 3.40-7.44; P < 0.00001, **Figure 3**). A random-effect model was used because significant heterogeneity in the five studies was found (Chi^2 ^= 15.71; df = 4 [p = 0.003]; I^2 ^= 75%) (**Figure 7**).

#### 3.3.5 R0 resection rate

**Figure 8:**
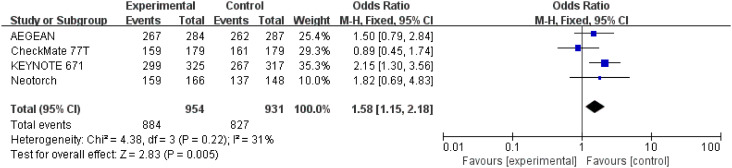
Assessment of R0 resection. The diamond indicates best estimate of the true (pooled) outcome (with width indicating 95% CI); OR, odds ratio; experimental stands for perioperative immunotherapy combined with chemotherapy; control stands for chemotherapy alone. Since there is moderate heterogeneity, a fixed-effects model is used.

Detailed data of R0 resection rate were extracted from four studies (26–29) involving a total 1,885 patients. The result indicated that perioperative immunotherapy plus chemotherapy was associated with significant benefit in R0 resection compared to chemotherapy alone (OR = 1.58; 95% CI: 1.15-2.18; P = 0.005). Moderate heterogeneity was found among the trials (Chi^2 ^= 4.38; df = 3 [p = 0.22]; I^2 ^= 31%) (**Figure 8**).

#### 3.3.6 Underwent surgery

**Figure 9:**
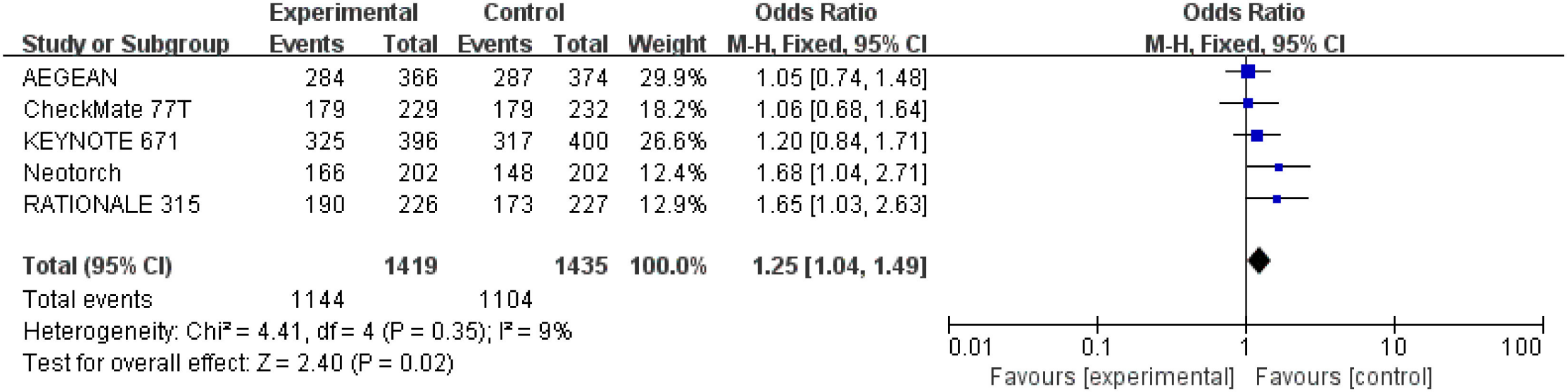
Assessment of rate of underwenting surgery. The diamond indicates best estimate of the true (pooled) outcome (with width indicating 95% CI); OR, odds ratio; experimental stands for perioperative immunotherapy combined with chemotherapy; control stands for chemotherapy alone. Since there is low heterogeneity, a fixed-effects model is used.

Detailed data of underwent surgery were extracted from five studies (26–30) involving a total 2,854 patients. Perioperative immunotherapy plus chemotherapy was associated with higher surgical resection rate compared to chemotherapy alone (OR = 1.25; 95% CI: 1.04-1.49; P=0.02) (**Figure 9**) Low heterogeneity was found among the trials (Chi^2 ^= 4.41; df = 4 [p= 0.35]; I^2 ^= 9%).

#### 3.3.7 Adverse events

Analysis of AEs showed that there was no statistical difference between perioperative immunotherapy plus chemotherapy and chemotherapy alone in the term of any treatment-related adverse event (TRAE) (OR = 1.52;95% CI: 0.95-2.45; P=0.08) and TRAE that led to dearth (OR = 1.12; 95% CI: 0.64-1.97; P=0.69). However, perioperative immunotherapy plus chemotherapy result in higher risk of grade 3+ TRAE (OR = 1.25;95% CI: 1.06-1.49; P=0.010), severe adverse event (SAE) (OR = 1.46;95% CI: 1.19-1.78; P=0.0002), TRAE that led to discontinuation of all trial treatment (OR = 1.90;95% CI: 1.34-2.68; P=0.0003), any iRAE (OR = 2.78;95% CI: 2.18-3.55; P<0.00001) and grade 3+ immune-related adverse event (iRAE) (OR = 2.89; 95% CI: 1.53-5.44; P=0.001) (**Table 2**; **Supplementary Figure 1**).

**Table 2:** Results of adverse events.

Toxicity	Odds Ratio	No. of trail	I^2^ statistic (%)
Any TRAE	1.52 [0.95, 2.45]	4	0
Grade 3 and higher TRAE	1.24 [1.06, 1.49]	5	19
Serious TRAE	1.46 [1.19, 1.78]	4	5
Any irAE	2.78 [2.18, 3.55]	2	0
Grade 3 and higher irAE	2.89 [1.53, 5.44]	2	36
TRAE that led to death	1.12 [0.64, 1.97]	3	0
TRAE that led to discontinuation of all trial treatment	1.90 [1.34, 2.68]	3	29

TRAE, treatment-related adverse event; irAE, immune-related adverse event.

### 3.4 Subgroup analysis

#### 3.4.1 Subgroup analysis of EFS

Data for subgroup analysis of EFS came from five trials. Overall, no differences were observed in subgroup analyses of age, sex, ECOG performance−status score, Stage of disease, lymph node station, histologic features, PD-L1 tumor proportion score, region, planned neoadjuvant platinum agent, pCR status and MPR status. However, we found differences in subgroup analysis of smoking status, EGFR status and pathological stage. Subgroup analysis showed significant survival benefit in current smoker (HR = 0.52;95% CI: 0.38-0.70), former smoker (HR = 0.63;95% CI: 0.52-0.76) and EGFR-mutation negative (HR = 0.55;95% CI: 0.45-0.66), but no in never smoked (HR = 0.76;95% CI: 0.52-1.12) and EGFR-mutation positive (HR = 0.35;95% CI: 0.04-3.03). Both II stage (HR = 0.66;95% CI: 0.51-0.86) and III stage (HR = 0.54;95% CI: 0.43-0.63) cloud benefit from perioperative immunotherapy plus chemotherapy. Further stratified analysis of stage III patients showed significant benefit in III A stage (HR = 0.55;95% CI: 0.47-0.66) and III B stage (HR = 0.54;95% CI: 0.32-0.92) (**Table 3**; **Supplementary Figures 2**-**4**).

**Table 3:** Results of subgroup analysis for event-free survival.

Sub-Group	No. of trail	I^2^ statistic (%)	Hazard ratio	P Value
Age				
<65 year old	4	0	0.54 [0.46, 0.65]	< 0.00001
≥65 year old	4	5	0.60 [0.50, 0.72]	< 0.00001
Sex				
male	4	29	0.56 [0.48, 0.64]	< 0.00001
female	4	28	0.64 [0.49, 0.83]	0.0010
Smoking status				
Current smoker	2	0	0.52 [0.38, 0.70]	< 0.0001
Former smoker	2	31	0.63 [0.52, 0.76]	< 0.00001
Never smoked	4	0	0.73 [0.51, 1.05]	0.09
ECOG performance-status score				
ECOG PS 0	3	0	0.58 [0.48, 0.71]	< 0.00001
ECOG PS 1	3	49	0.56 [0.44, 0.71]	< 0.00001
Patdological stage				
II	3	0	0.66 [0.51, 0.86]	0.002
III	4	15	0.54 [0.46, 0.63]	< 0.00001
IIIA	2	0	0.55 [0.47, 0.66]	< 0.00001
IIIB	2	84	0.54 [0.32, 0.92]	0.02
Lymph node station				
N2 single	2	0	0.56 [0.40, 0.78]	0.0007
N2 multi	2	0	0.54 [0.33, 0.90]	0.02
Histologic features				
Squamous	4	45	0.52 [0.40, 0.66]	< 0.00001
Non-squamous	4	0	0.64 [0.53, 0.77]	< 0.00001
PD-L1 expression at baseline				
PD-L1 TPS <1%	4	0	0.74 [0.61, 0.90]	0.003
PD-L1 TPS 1-49%	4	55	0.51 [0.37, 0.71]	< 0.0001
PD-L1 TPS ≥50%	4	37	0.45 [0.32, 0.62]	< 0.00001
Geographic region				
Asia	3	0	0.60 [0.46, 0.77]	< 0.0001
Non-Asia	3	21	0.62 [0.50, 0.76]	< 0.00001
Planned neoadjuvant platinum agent				
Cisplatin	3	0	0.59 [0.50, 0.71]	< 0.00001
Carboplatin	2	46	0.63 [0.46, 0.86]	0.004
pCR status				
pCR	2	0	0.33 [0.13, 0.86]	0.02
Non-pCR	2	0	0.72 [0.61, 0.86]	0.0003
MPR status				
MPR	2	0	0.48 [0.26, 0.86]	0.01
Non-MPR	2	0	0.77 [0.64, 0.93]	0.006
EGFR-mutation				
positive	2	72	0.35 [0.04, 3.03]	0.34
negative	4	37	0.55 [0.45, 0.66]	< 0.00001

ECOG, eastern cooperative oncology group; PS, performance-status score; PD-L1, programmed cell death-Ligand 1; TPS, tumor proportion score; pCR, pathological complete response; MPR, major pathological response; EGFR, epidermal growth factor receptor.

#### 3.4.2 Subgroup analysis of pCR

Data for subgroup analysis of pCR came from three trials. In general, no differences were observed in subgroup analyses of sex, smoking status, ECOG performance−status score, pathological stage, histologic features, PD-L1 tumor proportion score, region and planned neoadjuvant platinum agent (**Table 4**; **Supplementary Figures 5**, **6**).

**Table 4:** Results of subgroup analysis for pathological complete response.

Sub-Group	No. of trail	I^2^ statistic (%)	Odds ratio	P Value
Sex				
male	2	56	7.70 [4.21, 14.06]	< 0.00001
female	2	0	4.38 [1.94, 9.91]	0.0004
Smoking status				
Current/former smoker	2	59	7.10 [3.96, 12.74]	< 0.00001
Never smoked	2	0	6.19 [1.83, 21.00]	0.003
ECOG performance−status score				
ECOG PS 0	2	76	8.76 [1.04, 73.61]	0.05
ECOG PS 1	2	0	7.91 [4.36, 14.35]	< 0.00001
Patdological stage				
II	2	0	8.61 [4.65, 15.95]	< 0.00001
III	2	40	6.18 [3.51, 10.88]	< 0.00001
Histologic features				
Squamous	2	14	7.07 [4.43, 11.29]	< 0.00001
Non-squamous	2	0	6.52 [3.45, 12.34]	< 0.00001
PD-L1 expression at baseline				
PD-L1 TPS <1%	2	0	4.42 [2.41, 8.09]	< 0.00001
PD-L1 TPS 1-49%	2	16	5.10 [2.28, 11.38]	< 0.0001
PD-L1 TPS ≥50%	2	0	10.35 [4.70, 22.78]	< 0.00001
Geographic region				
Asia	2	61	9.45 [1.70, 52.53]	0.01
Non-Asia	2	0	4.76 [2.70, 8.40]	< 0.00001
Planned neoadjuvant platinum agent				
Cisplatin	2	0	7.25 [2.46, 21.37]	0.0003
Carboplatin	2	0	5.09 [3.15, 8.22]	< 0.00001

PD-L1, programmed cell death-Ligand 1; TPS, tumor proportion score.

### 3.5 Sensitivity analyses and publication bias

Sensitivity analysis via study-by-study removal showed that after removing Neotorch or RATIONALE-315, we found the stability of the results for R0 resection was compromised. Moreover, after removing, the stability of the results for rate of underwent surgery was also affected. However, the remaining efficacy endpoints remained stable. Qualitative assessment was performed by assessing various measures for each individual study using the Cochrane Risk of Bias Tool. In general, due to all trials were randomized, controlled, double-blind trials, they were considered to have low risk of bias. Funnel plot asymmetry was not obvious to any result (**Supplementary Figures 7**-**12**). Egger regression test results showed that EFS (0.322), MPR (0.068), R0 resection rate (0.327), rare of underwent surgery (0.220) had a low potential for publication bias, but pCR (0.008) and OS (0.039) had a significant publication bias.

## 4 Discussion

Resectable NSCLC mainly refers to stage I-II and some locally advanced (stage III) tumors (31). Surgery is the only radical treatment at present, but the recurrence rate is high, and perioperative treatment cannot significantly improve the survival prognosis of patients. So even if the tumor is surgically removed, many patients may require further therapeutic interventions. Recent studies showed that perioperative immunotherapy combined with chemotherapy can improve survival benefits in patients with resectable NSCLC. For example, the previous NADIM study (18) and CheckMate-816 study (16) both confirmed that neoadjuvant immunotherapy plus chemotherapy can improve survival prognosis of patients with resectable NSCLC compared with chemotherapy alone. Particularly, in CheckMate-816 (16), three cycles of neoadjuvant nivolumab plus chemotherapy without postoperative adjuvant immunotherapy improved pCR and EFS in patients with resectable stage IB-IIIB NSCLC. This therapeutic regimen did not hind the feasibility of surgery or increase the incidence of adverse events, but showed significant survival benefit. This combination regimen will be given a brighter future. In addition, immune checkpoint inhibitors (ICI) have also shown durable response rates in NSCLC, especially in squamous cell NSCLC. A combination of neoadjuvant immunotherapy and adjuvant immunotherapy could potentially be beneficial. Therefore, we pooled data on the efficacy and safety of perioperative immunotherapy combined with chemotherapy versus chemotherapy alone in resectable NSCLC, performing a meta-analysis to evaluate the efficacy and safety of perioperative immunotherapy combined with chemotherapy versus chemotherapy alone in the treatment of resectable NSCLC. The results indicated that perioperative immunotherapy plus chemotherapy versus chemotherapy alone significantly improved EFS, pCR, and OS in patients with resectable NSCLC. Previous meta-analysis by Marinelli et al. (32) showed that adding anti-PD(L)1 agents to neoadjuvant platinum-based chemotherapy led to improved prognosis in patients with resectable NSCLC. This meta-analysis also included patients who received neoadjuvant immunotherapy or perioperative immunotherapy. Moreover, phase II clinical studies are excluded in this meta-analysis. As we all know, the conclusions and data of phase II clinical studies are not mature and the level of evidence is not high. For example, Mobocertinib and Tiragolumab showed positive results in phase II clinical data (33, 34), but showed negative results in phase III clinical studies (35, 36). Our only included phase III clinical trials, excluding phase II clinical trials, to provide more direct and powerful evidence for the value of immunotherapy in perioperative treatment. Moreover, we also conducted subgroup analysis of EFS to further explore the influence of different factors, especially pCR status.

PCR is a predictor of long-term prognosis of neoadjuvant therapy (37, 38), which has been confirmed by some studies. Results from a research (39) showed that 5-year OS in patients who obtained pCR was 80% compared with those who did not obtain pCR, and the correlation between pCR and OS was statistically significant (P=0.0007). A retrospective study by Donington et al., which evaluated the relationship between EFS and OS in 221 patients with resectable stage II-III B (N2) NSCLC treated with neoadjuvant therapy, found a positive association between EFS and OS (0.68;95%CI: 0.52-0.79). Moreover, patients with recurrence were associated with a significantly shorter median OS (19.3 *vs*.116.9 months) and a 4.59-time increased risk of death (95%CI:2.56-8.26) compared with patients without recurrence (40). The results show that pCR and EFS can be used as alternative endpoints for survival benefit in patients with resectable NSCLC. It is worth noting that the five studies included in this meta-analysis all had EFS as their primary endpoint and all had positive results. In addition, different from the previous CheckMate-816, IMpower010 and KEYNOTE-091 studies, the OS of our meta-analysis showed statistical differences (HR = 0.68;95% CI: 0.56-0.83; P = 0.0002). So, the underlying trend in combination therapy in resectable non-small cell lung cancer is favorable, while data on overall survival require continued follow-up to mature. Besides, similar to the results of the Checkmate-816 trial, there is a higher proportion of patients achieving pCR after neoadjuvant ICI plus chemotherapy, and the patients have significantly longer EFS. Therefore, an important clinical question still remains whether adjuvant ICI monotherapy is necessary for patients who have not achieved pCR. However, it is worth noting that in the CheckMate-816 and CheckMate-77T trials, patients who did not achieve pCR all showed a trend of EFS benefit, but there was no statistical difference. A detailed study of the treatment regimen in CheckMate-816 revealed that it allowed patients to use adjuvant chemotherapy or radiotherapy, which confounded the accuracy of these results. Our meta-analysis also found significant EFS benefit for patients who did not achieve pCR after neoadjuvant immunotherapy. Additionally, although the data from CheckMate-816 showed a trend of benefit in OS, it was not statistically different. In conclusion, neoadjuvant immunotherapy plus chemotherapy alone may not achieve maximal benefit. In addition, the efficacy of adjuvant immunotherapy was explored in IMpower010 and KEYNOTE-091. The results showed the benefit of adjuvant immunotherapy in longer EFS, demonstrating the necessity of adjuvant immunotherapy. However, IMpower010 and KEYNOTE-091 did not show any OS benefit. Thus, it seems that adjuvant immunotherapy alone did not achieve maximum benefit. Based on the current data, there was no significant benefit in OS in CheckMate-816, Impower010, and KEYNOTE-091. Currently, KEYNOTE-671 study is the only one investigating neoadjuvant therapy for NSCLC with OS and EFS as primary endpoints, considering the significance of OS as the gold standard and the follow-up time cost associated with OS as an endpoint. This study also confirms significant benefits in EFS and OS with pembrolizumab around the perioperative period, both achieving statistical differences. Similarly, Neotorch and RATIONALE-315 both confirmed significant benefits of perioperative immunotherapy in EFS and OS. Our meta-analysis also showed similar results. Compared with neoadjuvant immunochemotherapy alone, additional adjuvant immunotherapy may further eliminate residual lesions and micrometastasis. Taken together, these data may suggest that some patients could benefit from adjuvant ICI monotherapy following neoadjuvant ICI plus chemotherapy and surgical resection. In addition, from the results of a subgroup analysis of KEYNOTE-671, it appears to be a trend of greater benefit in the population with stage III. It is crucial to select appropriate candidates for perioperative immunotherapy. In addition, further exploration is needed to determine whether continuing adjuvant immunotherapy is warranted based on pathological response. DNA sequencing technology may give us an answer. With the progress of DNA sequencing technology, ctDNA (circulating tumor DNA, ctDNA) can serve as an early detection tool for cancer, to a certain extent, enabling improved treatment outcomes through early intervention. We found higher ctDNA clearance in the combination-therapy group (56%) than in the chemotherapy-alone group (35%). And patients with ctDNA clearance had longer median EFS than those without ctDNA clearance, regardless of whether they were treated with combination therapy or chemotherapy alone, the percentage of ctDNA-cleared patients achieving pCR was also higher in both treatment groups than in the ctDNA-uncleared patients (41). However, ctDNA is a common indicator of MRD. Based on the above conclusions, we consider whether the detection of MRD can further predict the risk of tumor recurrence (42). Minimal residual disease (MRD) may be helpful for the treatment decision after surgery. MRD refers to the molecular abnormalities of cancer origin that cannot be detected by traditional imaging (including PET/CT) or laboratory methods after treatment, but can be found by liquid biopsy, which represents the persistence and clinical progression of lung cancer. Zhang et al. examined peripheral blood MRD from 261 stage I to III NSCLC patients who underwent radical surgery. The result showed that adjuvant therapy can significantly improve disease-free survival in MRD+ patients, but not in MRD+ patients (43). This means that MRD - patients have a very low tumor burden and that these patients may not require adjuvant therapy. In the longer term, MRD to guide the choice of treatment mode may become a research direction Identifying and avoiding overtreatment of a potentially curable population is an important clinical issue. Further studies are still needed to stratifying patients so as to identify those who require postoperative adjuvant immunotherapy.

Our meta-analysis performed a subgroup analysis to explore the effect of baseline characteristics on EFS. The result showed differences in subgroup of smoking status and pathological stage. Notably, significant benefits were observed across most subgroups of EFS and pCR. However, no statistical differences were observed for EFS of never smoked patient. We also found that men benefited more from perioperative immunotherapy than women in the term of EFS (P < 0.00001 *vs* P=0.00010). Firstly, it (44) has been shown that sex differences in the immune system play a key role in cancer. Because men and women are born with sex chromosome differences, sensitivity to combination therapy is also different. The Y chromosome is rich in repair genes, which are consistent with anti-inflammatory M2-type tumor-associated macrophages. M2-type tumor-associated macrophages are also associated with tumor immunosuppression and poor prognosis. Therefore, this may be one reason why female benefit less from the combination therapy. In addition, data from previous studies may also explain this phenomenon. Estrogen may promote resistance to vascular endothelial growth factor targeted therapy by increasing myeloid recruitment (45). Female patients have more estrogen in their bodies, resulting in higher resistance to our combination therapy, which affects their benefit. Moreover, due to economic, social and cultural factors, the proportion of never-smoking females is larger compared to males. Although cross-study comparisons should be made with caution considering in different designs and chemotherapy regimens, there is more overlap between women and never smokers, which also provides an explanation for the lack of benefit among non-smokers. In addition, as a complex substance, tobacco can not only cause cancer, but also increase the mutation load of tumors and produce more neoantigens, which is conducive to further cancer treatment. Studies showed that carcinogens in cigarettes increased PD-L1 levels. PD-L1 helped tumor cells escape T-cell recognition and promoted tumor development. After the occurrence of tumor, the expression level of PD-L1 in tumor cells was positively correlated with the effect of immunotherapy to some extent (46). So patients who smoke will have better effects of immunotherapy than those who have never smoked. Subgroup analyses of stage IIIB suggest significant heterogeneity. In the AEGEAN study, stage IIIB patients did not benefit from perioperative immunotherapy. One possible explanation is that AEGEAN study applied PD-L1 inhibitors, and the Neotorch and KEYNOTE 671 studies applied PD-1 inhibitors. It is worth noting that Neotorch received a postoperative course of adjuvant immunotherapy plus chemotherapy followed by maintenance immunotherapy, whereas AEGEAN received only adjuvant immunotherapy. The Neotorch study designed innovatively adopted a new model of “3 + 1 + 13” perioperative immunotherapy, that is, “3 cycles of toripalimab plus chemotherapy” as neoadjuvant therapy, adjuvant therapy with 1 cycle of toripalimab plus chemotherapy and consolidation therapy with 13 cycles of toripalimab. After 1 cycle of immunotherapy plus chemotherapy, the patients were given adjuvant immunotherapy. For patients with higher tumor burden, the number and probability of residual lesions after surgery appear to be higher. Besides, immunotherapy plus chemotherapy also eliminated residual tumor cells better than immunization alone. Therefore, for patients with a higher tumor burden, the treatment mode of Neotorch may be more beneficial, which is worth further research to explore. Additional chemotherapy after surgery may allow better clearance of residual disease. Different types of immune checkpoint inhibitors may be another possible reason. AEGEAN trial and Neotorch trial included many N2 patients and the heterogeneity of patients with stage III N2 NSCLC was high. In stage III N2 NSCLC, toripalimab (a PD-1 inhibitor) plus chemotherapy resulted in longer EFS, but durvalumab (a PD-L1 inhibitor) plus chemotherapy did not. But there is no universal standard of treatment. According to the NCCN and CSCO guidelines, even if N2 is surgically resectable, the guidelines still primarily recommend concurrent chemoradiotherapy. Therefore, whether perioperative immunotherapy combined with chemotherapy can bring more survival benefits to more patients needs to be further explored. Moreover, patients with stage III B and patients with stage III A seem to be treated similarly, but the latter has a greater survival benefit. This is something that we need to explore. Our meta-analysis separately indicated that patients with higher PD-L1 expression had more significant benefit from perioperative immunotherapy. HR of EFS for PD-L1 TPS <1%, 1–49% and ≥50% was 0.74, 0.51 and 0.45 separately. This suggested that PD-L1 expression may be a biomarker for predicting the efficacy of perioperative immunotherapy. Subgroup analysis of EGFR status suggested that there was no clear evidence of clinical benefit with the use of perioperative immunotherapy plus chemotherapy in patient with EGFR-mutation positive.

Regarding AEs, our meta-analysis showed that perioperative immunotherapy combined with chemotherapy did not impede the feasibility of surgery. This combination did not lead to higher risk of death and any grade TRAE. However, compared with chemotherapy alone, it increased SAE, grade 3 +TRAEs and the TRAE that led to treatment interruption. This is mainly due to the fact that immunotherapy attacks tumor cells by activating the immune system, during which the immune system may attack normal tissues and result in autoimmune reactive adverse events such as rash, gastrointestinal reaction, hepatotoxicity, nephrotoxicity and so on (47). Overall, immunotherapy resulted in more severe adverse events, so monitoring for adverse events is warranted.

Neoadjuvant immunotherapy combined with chemotherapy is currently a hot treatment for resectable NSCLC. Especially, studies such as CheckMate-816 and NADIM have opened up an era of neoadjuvant immunotherapy. Perioperative immunotherapy is expected to become a better choice for patients with resectable NSCLC. Our meta-analysis also performed subgroup analyses to explore the effect of pCR status on EFS. The result showed that patients with or without pCR could benefit from Perioperative immunotherapy plus chemotherapy. Interestingly, an exploratory analysis of CheckMate-816 showed a similar pattern. But, for non-pCR population after neoadjuvant immunotherapy, the EFS HR in Check Mate-816 was 0.84, while that in KEYNOTE-671 and Check Mate-77T was 0.69 and 0.79, respectively. The NEOTORCH study showed a very good EFS benefit curve for non-pCR population, although the HR value of EFS benefit has not been calculated. These results indicated that continuing adjuvant immunotherapy is expected to further improve the prognosis of patients without pCR after neoadjuvant immunotherapy. Although the 2-year EFS rates of patients who reached pCR in CheckMate-816, KEYNOTE-671, and NEOTORCH studies were more than 90%. However, in CheckMate-816, the EFS curve of patients who reached pCR began to decline after 30-40 months of follow-up, suggesting that patients with pCR after neoadjuvant immunotherapy may need to continue adjuvant immunotherapy to further improve their survival benefits. Nonetheless, there is a lack of head-to-head study of perioperative immunotherapy and neoadjuvant immunotherapy. So, it is not clear which patients need adjuvant immunotherapy, how long immunotherapy is optimal in the adjuvant phase and can benefit from it.

This review has several advantages that it conducts subgroup analysis of resectable NSCLC to explore the effect of baseline features on perioperative immunotherapy combined with chemotherapy. Besides, five of the included studies mentioned blinding and these trials were considered to have a lower risk bias in addition to detection bias as assessed by the Cochrane Bias Risk Tool. In order to eliminate the limitation of follow-up time, we summarized the data of EFS and pCR to try to replace OS in evaluating the efficacy of perioperative immunotherapy combined with chemotherapy. Of course, our study also has limitations. First, only 5 studies were included in this meta-analysis. Second, there were some differences in the treatment protocols of the included studies. Third, the follow-up time of most studies is insufficient, which makes it difficult to evaluate the effect of perioperative immunotherapy combined with chemotherapy on OS more comprehensively, so it may lead to certain bias.

## 5 Conclusions

This meta-analysis found superior pCR, MPR and EFS associated with perioperative immunotherapy combined with chemotherapy in resectable stage II-IIIB NSCLC. Although the OS data is still immature, containing only three studies, it also shows a trend of benefit. Perioperative immunotherapy plus chemotherapy can also improve the R0 resection rate and the rate of surgery, but the results need to be interpreted with caution due to unstable results. The application of adjuvant immunotherapy after neoadjuvant immunotherapy plus chemotherapy remains inconclusive due to the lack of head-to-head studies. Additional studies are needed to identify patients who require adjuvant therapy. Patient, tumor, and treatment factors should be considered when using perioperative immunotherapy, as individualized therapy is the current trend. Further confirmation is still needed.

## Data availability statement

The original contributions presented in the study are included in the article/**Supplementary Material**, further inquiries can be directed to the corresponding author.

## Author contributions

WZ: Writing – original draft, Formal analysis, Methodology, Validation, Visualization, Conceptualization, Data curation. ZL: Formal analysis, Methodology, Validation, Visualization, Writing – original draft, Conceptualization, Data curation. YZ: Formal analysis, Methodology, Validation, Visualization, Writing – review & editing. YL: Software, Writing – review & editing. TC: Project administration, Writing – review & editing. WL: Software, Writing – review & editing. YC: Project administration, Validation, Writing – review & editing. PW: Project administration, Validation, Writing – review & editing. HZ: Funding acquisition, Supervision, Writing – review & editing. CF: Writing – review & editing, Supervision, Validation. LL: Funding acquisition, Supervision, Writing – review & editing, Project administration, Validation, Visualization.

## References

[1] Molina JR Yang P Cassivi SD Schild SE Adjei AA . Non-small cell lung cancer: epidemiology, risk factors, treatment, and survivorship. Mayo Clin Proc. (2008) 83:584–94. doi: 10.4065/83.5.584 PMC2718421 18452692

[2] Aramini B Masciale V Samarelli AV Dubini A Gaudio M Stella F . Phenotypic, functional, and metabolic heterogeneity of immune cells infiltrating non-small cell lung cancer. Front Immunol. (2022) 13:959114. doi: 10.3389/fimmu.2022.959114 36032082 PMC9399732

[3] Liu B Ding F Yang S . Progress of postoperative adjuvant chemotherapy in stage I non-small cell lung cancer. Zhongguo Fei Ai Za Zhi. (2015) 18:374–80. doi: 10.3779/j.issn.1009-3419.2015.06.08 PMC5999912 26104895

[4] Blandin Knight S Crosbie PA Balata H Chudziak J Hussell T Dive C . Progress and prospects of early detection in lung cancer. Open Biol. (2017) 7:170070. doi: 10.1098/rsob.170070 28878044 PMC5627048

[5] Van Schil PE . Surgery: therapeutic indications. Cancer Radiother. (2007) 11:47–52. doi: 10.1016/j.canrad.2006.06.001 16837227

[6] Rothschild SI Zippelius A Eboulet EI Savic Prince S Betticher D Bettini A . SAKK 16/14: durvalumab in addition to neoadjuvant chemotherapy in patients with stage IIIA(N2) non-small-cell lung cancer-A multicenter single-arm phase II trial. J Clin Oncol. (2021) 39:2872–80. doi: 10.1200/JCO.21.00276 34251873

[7] Indini A Rijavec E Bareggi C Grossi F . Novel treatment strategies for early-stage lung cancer: the oncologist's perspective. J Thorac Dis. (2020) 12:3390–8. doi: 10.21037/jtd.2020.02.46 PMC7330760 32642264

[8] Pless M Stupp R Ris HB Stahel RA Weder W Thierstein S . Induction chemoradiation in stage IIIA/N2 non-small-cell lung cancer: a phase 3 randomized trial. Lancet. (2015) 386:1049–56. doi: 10.1016/S0140-6736(15)60294-X 26275735

[9] Xu YP Li B Xu XL Mao WM . Is there a survival benefit in patients with stage IIIA (N2) non-small cell lung cancer receiving neoadjuvant chemotherapy and/or radiotherapy prior to surgical resection: A systematic review and meta-analysis. Med (Baltimore). (2015) 94:e879. doi: 10.1097/MD.0000000000000879 PMC4616485 26061306

[10] Hirsch FR Scagliotti GV Mulshine JL Kwon R Jr Curran WJ Wu YL . Lung cancer: current therapies and new targeted treatments. Lancet. (2017) 389:299–311. doi: 10.1016/S0140-6736(16)30958-8 27574741

[11] Musika W Kamsa-Ard S Jirapornkul C Santong C Phunmanee A . Lung cancer survival with current therapies and new targeted treatments: A comprehensive update from the srinagarind hospital-based cancer registry from (2013 to 2017). Asian Pac J Cancer Prev. (2021) 22:2501–7. doi: 10.31557/APJCP.2021.22.8.2501 PMC8629471 34452564

[12] Ren Y Tang H Zhang J She Y Sun X Xie D . Bayesian network meta-analysis of efficacy and safety of neoadjuvant therapy for non-small-cell lung cancer. Ther Adv Med Oncol. (2020) 12:1758835920973567. doi: 10.1177/1758835920973567 33240402 PMC7675866

[13] Yin C Hu B Yang X Kou L Tian B Wang C . Neoadjuvant sintilimab combined with chemotherapy in resectable locally advanced non-small cell lung cancer: case series and literature review. World J Surg Oncol. (2023) 21:304. doi: 10.1186/s12957-023-03194-4 37749594 PMC10521519

[14] Shalata W Yakobson A Dudnik Y Swaid F Ahmad MS Abu Jama A . Multi-center real-world outcomes of nivolumab plus ipilimumab and chemotherapy in patients with metastatic non-small-cell lung cancer. Biomedicines. (2023) 11:2438. doi: 10.3390/biomedicines11092438 37760878 PMC10525289

[15] Spigel DR Vicente D Ciuleanu TE Gettinger S Peters S Horn L . Second-line nivolumab in relapsed small-cell lung cancer: CheckMate 331☆. Ann Oncol. (2021) 32:631–41. doi: 10.1016/j.annonc.2021.01.071 33539946

[16] Forde PM Spicer J Lu S Provencio M Mitsudomi T Awad MM . Neoadjuvant nivolumab plus chemotherapy in resectable lung cancer. N Engl J Med. (2022) 386:1973–85. doi: 10.1056/NEJMoa2202170 PMC9844511 35403841

[17] Felip E Altorki N Zhou C Csőszi T Vynnychenko I Goloborodko O . Adjuvant atezolizumab after adjuvant chemotherapy in resected stage IB-IIIA non-small-cell lung cancer (IMpower010): a randomized, multicentre, open-label, phase 3 trial. Lancet. (2021) 398:1344–57. doi: 10.1016/S0140-6736(21)02098-5 34555333

[18] Provencio M Serna-Blasco R Nadal E Insa A García-Campelo MR Casal Rubio J . Overall Survival and Biomarker Analysis of Neoadjuvant Nivolumab Plus Chemotherapy in Operable Stage IIIA Non-Small-Cell Lung Cancer (NADIM phase II trial). J Clin Oncol. (2022) 40:2924–33. doi: 10.1200/JCO.21.02660 PMC9426809 35576508

[19] Nuccio A Viscardi G Salomone F Servetto A Venanzi FM Riva ST . Systematic review and meta-analysis of immune checkpoint inhibitors as single agent or in combination with chemotherapy in early-stage non-small cell lung cancer: Impact of clinicopathological factors and indirect comparison between treatment strategies. Eur J Cancer. (2023) 195:113404. doi: 10.1016/j.ejca.2023.113404 37948842 PMC12697757

[20] Ramos-Casals M Brahmer JR Callahan MK Flores-Chávez A Keegan N Khamashta MA . Immune-related adverse events of checkpoint inhibitors. Nat Rev Dis Primers. (2020) 6:38. doi: 10.1038/s41572-020-0160-6 32382051 PMC9728094

[21] Liberati A Altman DG Tetzlaff J Mulrow C Gøtzsche PC Ioannidis JP . The PRISMA statement for reporting systematic reviews and meta-analyses of studies that evaluate healthcare interventions: explanation and elaboration. BMJ. (2009) 339:b2700. doi: 10.1136/bmj.b2700 19622552 PMC2714672

[22] Higgins JP Altman DG Gøtzsche PC Jüni P Moher D Oxman AD . The Cochrane Collaboration's tool for assessing risk of bias in randomised trials. BMJ. (2011) 343:d5928. doi: 10.1136/bmj.d5928 22008217 PMC3196245

[23] Higgins JP Thompson SG Deeks JJ Altman DG . Measuring inconsistency in meta-analyses. BMJ. (2003) 327:557–60. doi: 10.1136/bmj.327.7414.557 PMC192859 12958120

[24] Higgins JP Thompson SG . Quantifying heterogeneity in a meta-analysis. Stat Med. (2002) 21:1539–58. doi: 10.1002/sim.1186 12111919

[25] Egger M Davey Smith G Schneider M Minder C . Bias in meta-analysis detected by a simple, graphical test. BMJ. (1997) 315:629–34. doi: 10.1136/bmj.315.7109.629 PMC2127453 9310563

[26] Heymach JV Mitsudomi T Harpole D Aperghis M Jones S Mann H . Design and rationale for a phase III, double-blind, placebo-controlled study of neoadjuvant durvalumab + Chemotherapy followed by adjuvant durvalumab for the treatment of patients with resectable stages II and III non-small-cell lung cancer: the AEGEAN trial. Clin Lung Cancer. (2022) 23:e247–51. doi: 10.1016/j.cllc.2021.09.010 34819266

[27] Cascone T Awad MM Spicer JD He J Lu S Sepesi B . LBA1 CheckMate 77T: Phase III study comparing neoadjuvant nivolumab (NIVO) plus chemotherapy (chemo) vs neoadjuvant placebo plus chemo followed by surgery and adjuvant NIVO or placebo for previously untreated, resectable stage II–IIIb NSCLC. Ann Oncol. (2023) 34:S1295. doi: 10.1016/j.annonc.2023.10.050

[28] Ruck JM Broderick SR . Neoadjuvant and adjuvant pembrolizumab for the treatment of early-stage resectable non-small cell lung cancer an editorial regarding the interim data analysis of the KEYNOTE-671 phase III trial of neoadjuvant and adjuvant pembrolizumab. Ann Surg Oncol. (2024) 31:4–5. doi: 10.1245/s10434-023-14356-9 37884698

[29] Lu S Wu L Zhang W Zhang P Wang W Fang W . Perioperative toripalimab + platinum-doublet chemotherapy vs chemotherapy in resectable stage II/III non-small cell lung cancer (NSCLC): Interim event-free survival (EFS) analysis of the phase III NEOTORCH study. J Clin Oncol. (2023) 41:8501. doi: 10.1200/JCO.2023.41.16suppl.8501

[30] Yue D Wang W Liu H Chen Q Chen C Zhang J . LBA58 Pathological response to neoadjuvant tislelizumab (TIS) plus platinum-doublet (PtDb) chemotherapy (CT) in resectable stage II-IIIA NSCLC patients (pts) in the phase III (Ph3) RATIONALE-315 trial. Ann Oncol. (2023) 34:S1299. doi: 10.1016/j.annonc.2023.10.054

[31] Liu X Xing H Liu H Chen J . Current status and future perspectives on immunotherapy in neoadjuvant therapy of resectable non-small cell lung cancer. Asia Pac J Clin Oncol. (2022) 18:335–43. doi: 10.1111/ajco.13665 34811893

[32] Marinelli D Gallina FT Pannunzio S Di Civita MA Torchia A Giusti R . Surgical and survival outcomes with perioperative or neoadjuvant immune-checkpoint inhibitors combined with platinum-based chemotherapy in resectable NSCLC: A systematic review and meta-analysis of randomized clinical trials. Crit Rev Oncol Hematol. (2023) 192:104190. doi: 10.1016/j.critrevonc.2023.104190 37871779

[33] Zhou C Ramalingam SS Kim TM Kim SW Yang JC Riely GJ . Treatment outcomes and safety of mobocertinib in platinum-pretreated patients with EGFR exon 20 insertion-positive metastatic non-small cell lung cancer: A phase 1/2 open-label nonrandomized clinical trial. JAMA Oncol. (2021) 7:e214761. doi: 10.1001/jamaoncol.2021.4761 34647988 PMC8517885

[34] Jänne PA Wu Y-L Kato T Besse B Peters S Nguyen D . Mobocertinib (TAK-788) as first-line treatment vs platinum-based chemotherapy (CT) for NSCLC with EGFR exon 20 insertions (exon20ins). Ann Oncol. (2020) 31:S892–3. doi: 10.1016/jannonc.2020.08.1726

[35] Cho BC Abreu DR Hussein M Cobo M Patel AJ Secen N . Tiragolumab plus atezolizumab versus placebo plus atezolizumab as a first-line treatment for PD-L1-selected non-small-cell lung cancer (CITYSCAPE): primary and follow-up analyses of a randomised, double-blind, phase 2 study. Lancet Oncol. (2022) 23:781–92. doi: 10.1016/S1470-2045(22)00226-1 35576957

[36] Rudin CM Liu SV Soo RA Lu S Hong MH Lee JS . SKYSCRAPER-02: tiragolumab in combination with atezolizumab plus chemotherapy in untreated extensive-stage small-cell lung cancer. J Clin Oncol. (2024) 42:324–35. doi: 10.1200/JCO.23.01363 PMC10824371 37976444

[37] Pöttgen C Stuschke M Graupner B Theegarten D Gauler T Jendrossek V . Prognostic model for long-term survival of locally advanced non-small-cell lung cancer patients after neoadjuvant radiochemotherapy and resection integrating clinical and histopathologic factors. BMC Cancer. (2015) 15:363. doi: 10.1186/s12885-015-1389-4 25943191 PMC4428235

[38] von Minckwitz G Fontanella C . Comprehensive review on the surrogate endpoints of efficacy proposed or hypothesized in the scientific community today. J Natl Cancer Inst Monogr. (2015) 2015:29–31. doi: 10.1093/jncimonographs/lgv007 26063882

[39] Mouillet G Monnet E Milleron B Puyraveau M Quoix E David P . Pathologic complete response to preoperative chemotherapy predicts cure in early-stage non-small-cell lung cancer: combined analysis of two IFCT randomized trials. J Thorac Oncol. (2012) 7:841–9. doi: 10.1097/JTO.0b013e31824c7d92 22722786

[40] Donington J Hu X Zhang S Song Y Arunachalam A Chirovsky D . Event-free survival as a predictor of overall survival and recurrence burden of patients with non-small cell lung cancer receiving neoadjuvant therapy. J Thorac Cardiovasc Surg. (2023) 12:S0022-5223(23)01193-5. doi: 10.1016/j.jtcvs.2023.12.006 38092284

[41] Abbosh C Birkbak NJ Swanton C . Early Stage NSCLC - challenges to implementing ctDNA-based screening and MRD detection. Nat Rev Clin Oncol. (2018) 15:577–86. doi: 10.1038/s41571-018-0058-3 29968853

[42] Dou S Xie H Yang L . Chinese expert consensus on standards of PD-L1 immunohistochemistry testing for non-small cell lung cancer. Chinese Journal of Lung Cancer. (2021) 24(12):862–6. doi: 10.3779/j.issn.1009-3419.2021.102.44 PMC8695239 34743498

[43] Zhang JT Liu SY Gao W Liu SM Yan HH Ji L . Longitudinal undetectable molecular residual disease defines potentially cured population in localized non-small cell lung cancer. Cancer Discovery. (2022) 12:1690–701. doi: 10.1158/2159-8290.CD-21-1486 PMC9394392 35543554

[44] Clocchiatti A Cora E Zhang Y Dotto GP . Sexual dimorphism in cancer. Nat Rev Cancer. (2016) 16:330–9. doi: 10.1038/nrc.2016.30 27079803

[45] Patel SA Herynk MH Cascone T Saigal B Nilsson MB Tran H . Estrogen promotes resistance to bevacizumab in murine models of NSCLC. J Thorac Oncol. (2021) 16:2051–64. doi: 10.1016/j.jtho.2021.07.007 PMC10324274 34311109

[46] Wang GZ Zhang L Zhao XC Gao SH Qu LW Yu H . Author Correction: The Aryl hydrocarbon receptor mediates tobacco-induced PD-L1 expression and is associated with response to immunotherapy. Nat Commun. (2022) 13:3575. doi: 10.1038/s41467-022-30871-x 35732635 PMC9217980

[47] Das S Johnson DB . Immune-related adverse events and anti-tumor efficacy of immune checkpoint inhibitors. J Immunother Cancer. (2019) 7:306. doi: 10.1186/s40425-019-0805-8 31730012 PMC6858629

