# Supplementary material for: Efficacy and safety of neoadjuvant immunotherapy plus chemotherapy followed by adjuvant immunotherapy in resectable non-small cell lung cancer: a meta-analysis of phase 3 clinical trials
Source: Front Immunol. 2024 Apr 5;15:1359302. doi: 10.3389/fimmu.2024.1359302 (PMC11026587; doi:10.3389/fimmu.2024.1359302)
Supplement: Supplementary file 1 [file DataSheet_1.docx]

***Supplementary***

Context:

**eFigure. 1** Assessment of adverse event

**eFigure. 2** Results of subgroup analysis of event-free survival (1)

**eFigure. 3** Results of subgroup analysis of event-free survival (2)

**eFigure. 4** Results of subgroup analysis of event-free survival (3)

**eFigure. 5** Results of subgroup analysis of pathological complete response (1)

**eFigure. 6** Results of subgroup analysis of pathological complete response (2)

**eFigure. 7** Funnel plot of overall survival.

**eFigure. 8** Funnel plot of event-free survival.

**eFigure. 9** Funnel plot of pathological complete response

**eFigure. 10** Funnel plot of major pathological response

**eFigure. 11** Funnel plot of R0 resection rate

**eFigure. 12** Funnel plot of rate of underwenting surgery

**eMethods.** Search Strategy

**eFigure. 1** Assessment of adverse event


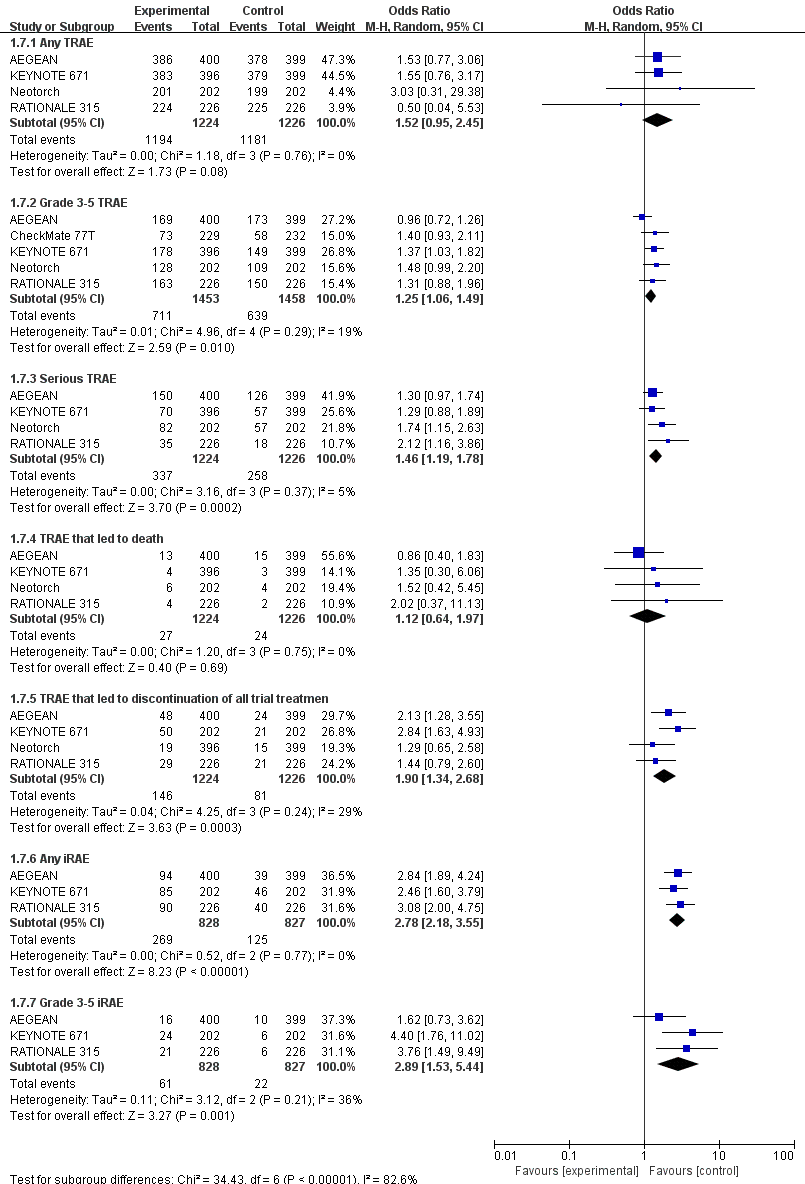


Abbreviations: The diamond indicates best estimate of the true (pooled) outcome (with width indicating 95% CI); OR, odds ratio; experimental stands for perioperative immunotherapy combined with chemotherapy; control stands for chemotherapy alone. Since there is moderate heterogeneity, a random-effects model is used.

**eFigure. 2** Results of subgroup analysis of event-free survival (1)


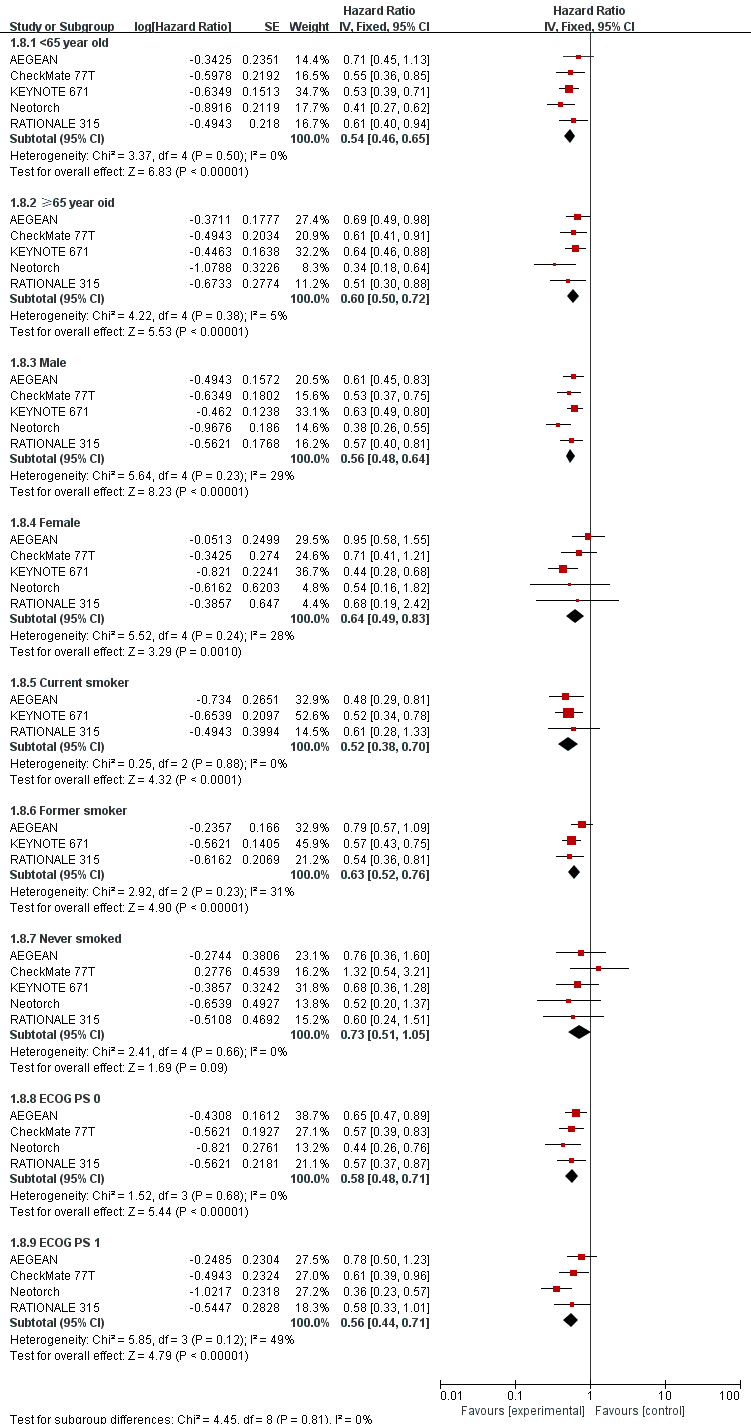


Abbreviations: The diamond indicates best estimate of the true (pooled) outcome (with width indicating 95% CI); HR, hazard ratio; experimental stands for perioperative immunotherapy combined with chemotherapy; control stands for chemotherapy alone. Since there is no heterogeneity, a random-effects model is used.

**eFigure. 3** Results of subgroup analysis of event-free survival (2)


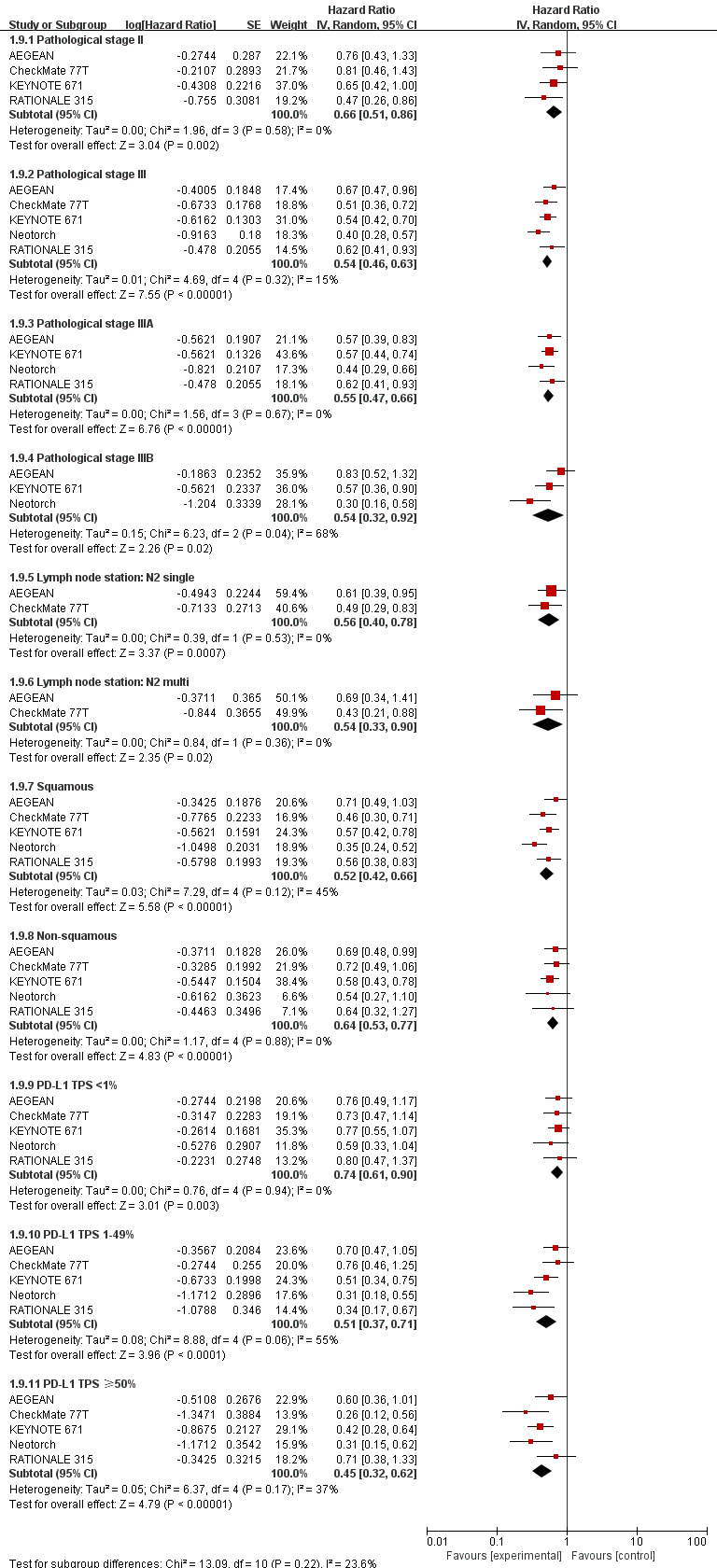


Abbreviations: The diamond indicates best estimate of the true (pooled) outcome (with width indicating 95% CI); HR, hazard ratio; experimental stands for perioperative immunotherapy combined with chemotherapy; control stands for chemotherapy alone. Since there is no heterogeneity, a random-effects model is used.

**eFigure 4.** Results of subgroup analysis of event-free survival (3)


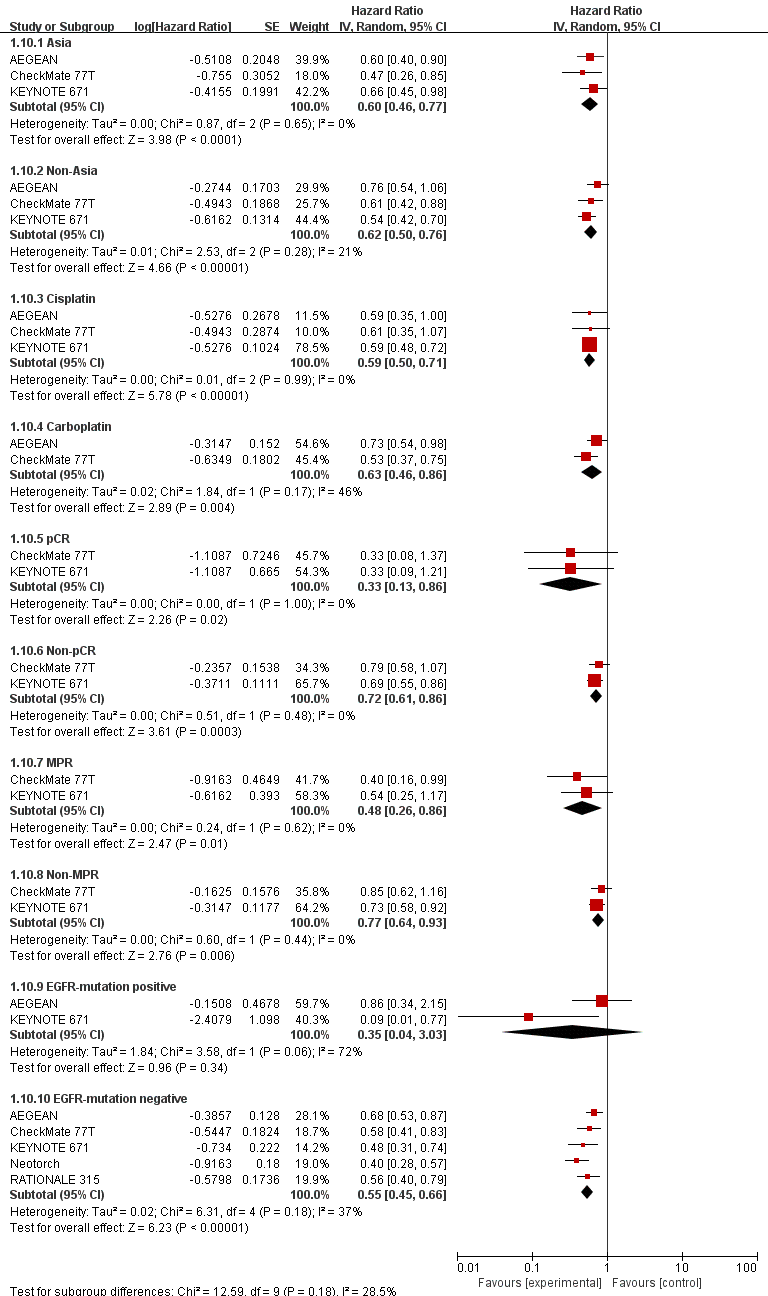


Abbreviations: The diamond indicates best estimate of the true (pooled) outcome (with width indicating 95% CI); HR, hazard ratio; experimental stands for perioperative immunotherapy combined with chemotherapy; control stands for chemotherapy alone. Since there is no heterogeneity, a random-effects model is used.

**eFigure 5.** Results of subgroup analysis of pathological complete response (1)


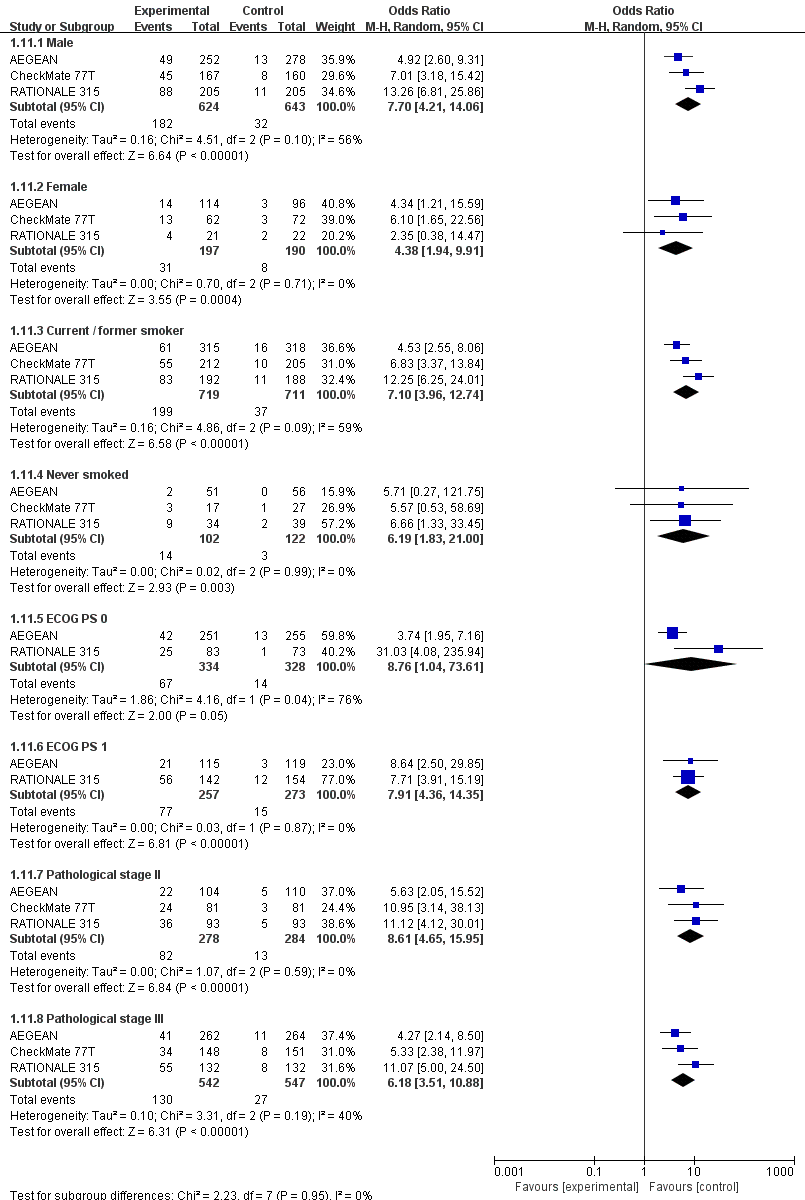


Abbreviations: The diamond indicates best estimate of the true (pooled) outcome (with width indicating 95% CI); OR, odds ratio; experimental stands for perioperative immunotherapy combined with chemotherapy; control stands for chemotherapy alone. Since there is moderate heterogeneity, a random-effects model is used.

**eFigure 6.** Results of subgroup analysis of pathological complete response (2)


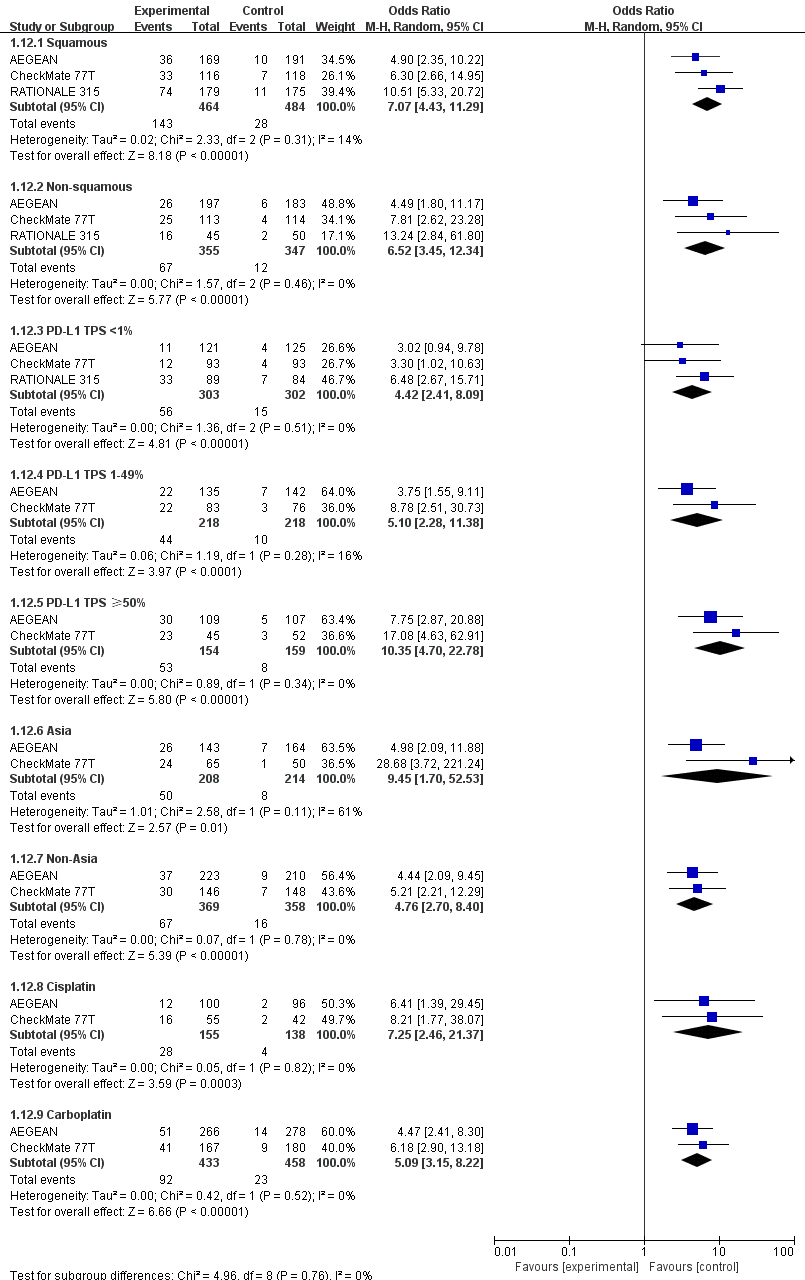


Abbreviations: The diamond indicates best estimate of the true (pooled) outcome (with width indicating 95% CI); OR, odds ratio; experimental stands for perioperative immunotherapy combined with chemotherapy; control stands for chemotherapy alone. Since there is moderate heterogeneity, a random-effects model is used.

**eFigure 7.** Funnel plot of overall survival.


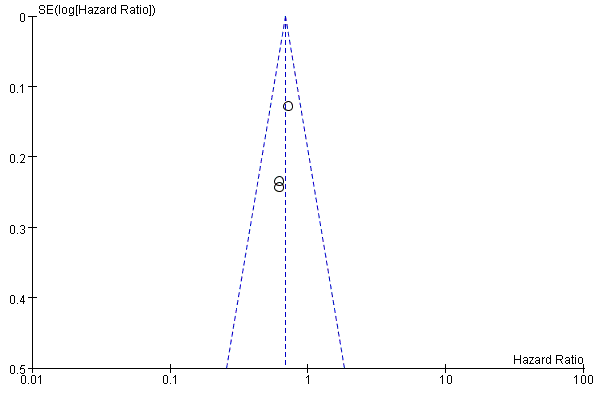


**eFigure 8.** Funnel plot of event-free survival.

**
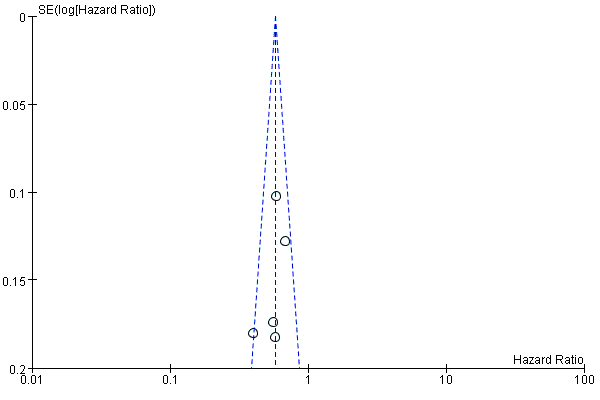
**

**eFigure. 9** Funnel plot of pathological complete response


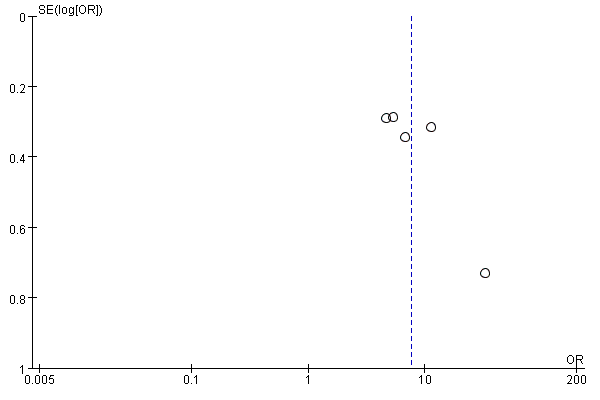


**eFigure. 10** Funnel plot of major pathological response


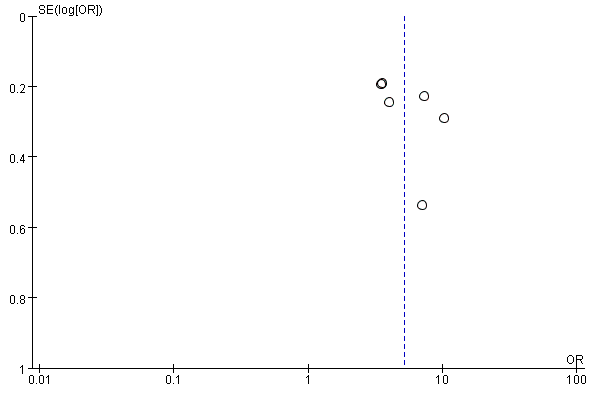


**eFigure. 11** Funnel plot of R0 resection rate


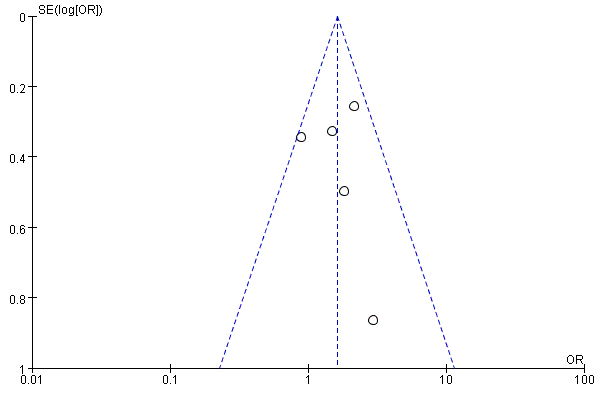


**eFigure. 12** Funnel plot of rate of underwenting surgery


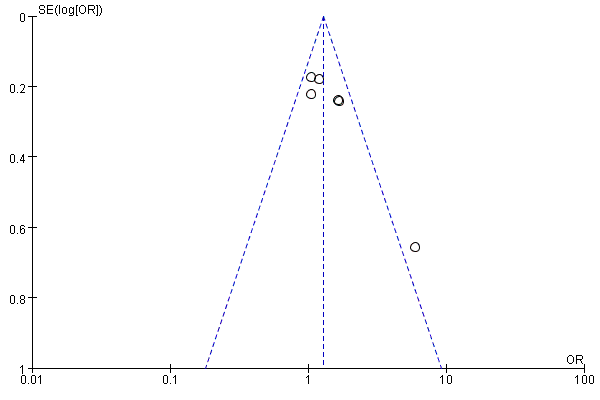


**eMethods.** Search Strategy

**PubMed < updated to 2023-11-01> (42)**

#1 Perioperative Period"[Mesh]

#2 Perioperative

#3 #1 OR #2

#4 "Immunotherapy"[Mesh]

#5 "Immune Checkpoint Inhibitors"[Mesh]

#6 Keytruda[Title/Abstract] OR Pembrolizumab[Title/Abstract] OR Opdivo[Title/Abstract] OR nivolumab[Title/Abstract] OR Imfinzi[Title/Abstract] OR Durvalumab[Title/Abstract] OR DHH02206[Title/Abstract] OR Tislelizumab[Title/Abstract] OR Toripalimab[Title/Abstract] OR ShuglizumabOR Sintilimab[Title/Abstract] OR Ipilimumab[Title/Abstract] OR Checkpoint Inhibitors, Immune[Title/Abstract] OR Immune Checkpoint Inhibitor[Title/Abstract] OR Checkpoint Inhibitor, Immune[Title/Abstract] OR Immune Checkpoint Blockers[Title/Abstract] OR Checkpoint Blockers, Immune[Title/Abstract] OR Immune Checkpoint Blockade[Title/Abstract] OR Checkpoint Blockade, Immune[Title/Abstract] OR Immune Checkpoint Inhibition[Title/Abstract] OR Checkpoint Inhibition, Immune[Title/Abstract] OR PD-L1 Inhibitors[Title/Abstract] OR PD L1 Inhibitors[Title/Abstract] OR PD-L1 Inhibitor[Title/Abstract] OR PD L1 Inhibitor[Title/Abstract] OR Programmed Death-Ligand 1 Inhibitors[Title/Abstract] OR Programmed Death Ligand 1 Inhibitors[Title/Abstract] OR PD-1-PD-L1 Blockade[Title/Abstract] OR Blockade, PD-1-PD-L1[Title/Abstract] OR PD 1 PD L1 Blockade[Title/Abstract] OR CTLA-4 Inhibitors[Title/Abstract] OR CTLA 4 Inhibitors[Title/Abstract] OR CTLA-4 Inhibitor[Title/Abstract] OR CTLA 4 Inhibitor[Title/Abstract] OR Cytotoxic T-Lymphocyte-Associated Protein 4 Inhibitors[Title/Abstract] OR Cytotoxic T Lymphocyte Associated Protein 4 Inhibitors[Title/Abstract] OR Cytotoxic T-Lymphocyte-Associated Protein 4 Inhibitor[Title/Abstract] OR Cytotoxic T Lymphocyte Associated Protein 4 Inhibitor[Title/Abstract] OR PD-1 Inhibitors[Title/Abstract] OR PD 1 Inhibitors[Title/Abstract] OR PD-1 Inhibitor[Title/Abstract] OR Inhibitor, PD-1[Title/Abstract] OR PD 1 Inhibitor[Title/Abstract] OR Programmed Cell Death Protein 1 Inhibitor[Title/Abstract] OR Programmed Cell Death Protein 1 Inhibitors[Title/Abstract]

#7 #4 OR #5 OR #6

#8 "Drug Therapy"[Mesh]

#9 Therapy, Drug[Title/Abstract] OR Drug Therapies[Title/Abstract] OR Therapies, Drug[Title/Abstract] OR Chemotherapy[Title/Abstract] OR Chemotherapies[Title/Abstract] OR Pharmacotherapy[Title/Abstract] OR Pharmacotherapies[Title/Abstract]

#10 #8 OR #9

#11 "Carcinoma, Non-Small-Cell Lung"[Mesh]

#12 Carcinomas, Non-Small-Cell Lung[Title/Abstract] OR Lung Carcinoma, Non-Small-Cell[Title/Abstract] OR Lung Carcinomas, Non-Small-Cell[Title/Abstract] OR Non-Small-Cell Lung Carcinomas[Title/Abstract] OR Non-Small-Cell Lung Carcinoma[Title/Abstract] OR NonSmall Cell Lung Carcinoma[Title/Abstract] OR Carcinoma, Non-Small Cell Lung[Title/Abstract] OR Non-Small Cell Lung Carcinoma[Title/Abstract] OR Non-Small Cell Lung Cancer[Title/Abstract] OR Nonsmall Cell Lung Cancer[Title/Abstract]

#13 #11 OR #12

#14 Early Stage[Title/Abstract] OR resectable[Title/Abstract]

#15 #3 AND #7 AND #10 AND #13 AND #14

**Embase < updated to 2023-11-01> (184)**

#1 'perioperative period'/exp

#2 perioperative:ab,ti

#3 #1 OR #2

#4 'immunotherapy'/exp

#5 'immune checkpoint inhibitor'/exp

#6 keytruda:ab,ti OR pembrolizumab:ab,ti OR opdivo:ab,ti OR nivolumab:ab,ti OR 'imfinzior durvalumab':ab,ti OR dhh02206:ab,ti OR 'tislelizumabor toripalimab':ab,ti OR 'shuglizumabor sintilimab':ab,ti OR ipilimumab:ab,ti OR 'checkpoint inhibitors, immune':ab,ti OR 'immune checkpoint inhibitor':ab,ti OR 'checkpoint inhibitor, immune':ab,ti OR 'immune checkpoint blockers':ab,ti OR 'checkpoint blockers, immune':ab,ti OR 'immune checkpoint blockade':ab,ti OR 'checkpoint blockade, immune':ab,ti OR 'immune checkpoint inhibition':ab,ti OR 'checkpoint inhibition, immune':ab,ti OR 'pd-l1 inhibitors':ab,ti OR 'pd l1 inhibitors':ab,ti OR 'pd-l1 inhibitor':ab,ti OR 'pd l1 inhibitor':ab,ti OR 'programmed death-ligand 1 inhibitors':ab,ti OR 'programmed death ligand 1 inhibitors':ab,ti OR 'pd-1-pd-l1 blockade':ab,ti OR 'blockade, pd-1-pd-l1':ab,ti OR 'pd 1 pd l1 blockade':ab,ti OR 'ctla-4 inhibitors':ab,ti OR 'ctla 4 inhibitors':ab,ti OR 'ctla-4 inhibitor':ab,ti OR 'ctla 4 inhibitor':ab,ti OR 'cytotoxic t-lymphocyte-associated protein 4 inhibitors':ab,ti OR 'cytotoxic t lymphocyte associated protein 4 inhibitors':ab,ti OR 'cytotoxic t-lymphocyte-associated protein 4 inhibitor':ab,ti OR 'cytotoxic t lymphocyte associated protein 4 inhibitor':ab,ti OR 'pd-1 inhibitors':ab,ti OR 'pd 1 inhibitors':ab,ti OR 'pd-1 inhibitor':ab,ti OR 'inhibitor, pd-1':ab,ti OR 'pd 1 inhibitor':ab,ti OR 'programmed cell death protein 1 inhibitor':ab,ti OR 'programmed cell death protein 1 inhibitors':ab,ti

#7 #4 OR #5 OR #6

#8 'chemotherapy'/exp

#9 'therapy, drug':ab,ti OR 'drug therapies':ab,ti OR 'therapies, drug':ab,ti OR chemotherapy:ab,ti OR chemotherapies:ab,ti OR pharmacotherapy:ab,ti OR pharmacotherapies:ab,ti

#10 #8 OR #9

#11 'non small cell lung cancer'/exp

#12 'carcinomas, non-small-cell lung':ab,ti OR 'lung carcinoma, non-small-cell':ab,ti OR 'lung carcinomas, non-small-cell':ab,ti OR 'non-small-cell lung carcinomas':ab,ti OR 'non-small-cell lung carcinoma':ab,ti OR 'nonsmall cell lung carcinoma':ab,ti OR 'carcinoma, non-small cell lung':ab,ti OR 'non-small cell lung carcinoma':ab,ti OR 'non-small cell lung cancer':ab,ti OR 'nonsmall cell lung cancer':ab,ti

#13 #11 OR #12

#14 'early stage':ab,ti OR resectable:ab,ti OR operable:ab,ti

#15 #3 AND #7 AND #10 AND #13 AND #14

**Cochrane library < updated to 2023-11-01> (4)**

#1 MeSH descriptor: [Perioperative Period] explode all trees

#2 (Perioperative):ti,ab,kw

#3 #1 OR #2

#4 MeSH descriptor: [Immunotherapy] explode all trees

#5 MeSH descriptor: [Immune Checkpoint Inhibitors] explode all trees

#6 (Keytruda OR Pembrolizumab OR Opdivo OR nivolumab OR Imfinzi OR Durvalumab OR DHH02206 OR Tislelizumab OR Toripalimab OR Shuglizumab OR Sintilimab OR Ipilimumab OR Checkpoint Inhibitors, Immune OR Immune Checkpoint Inhibitor OR Checkpoint Inhibitor, Immune OR Immune Checkpoint Blockers OR Checkpoint Blockers, Immune OR Immune Checkpoint Blockade OR Checkpoint Blockade, Immune OR Immune Checkpoint Inhibition OR Checkpoint Inhibition, Immune OR PD L1 Inhibitors OR PD L1 Inhibitors OR PD L1 Inhibitor OR PD L1 Inhibitor OR Programmed Death-Ligand 1 Inhibitors OR Programmed Death Ligand 1 Inhibitors OR CTLA 4 Inhibitors OR CTLA 4 Inhibitors OR CTLA 4 Inhibitor OR CTLA 4 Inhibitor OR Cytotoxic T Lymphocyte Associated Protein 4 Inhibitors OR Cytotoxic T Lymphocyte Associated Protein 4 Inhibitors OR Cytotoxic T Lymphocyte-Associated Protein 4 Inhibitor OR Cytotoxic T Lymphocyte Associated Protein 4 Inhibitor OR PD 1 Inhibitors OR PD 1 Inhibitors OR PD 1 Inhibitor OR Inhibitor, PD 1 OR PD 1 Inhibitor OR Programmed Cell Death Protein 1 Inhibitor OR Programmed Cell Death Protein 1 Inhibitors):ti,ab,kw

#7 #4 OR #5 OR #6

#8 MeSH descriptor: [Drug Therapy] explode all trees

#9 (Therapy, Drug OR Drug Therapies OR Therapies, Drug OR Chemotherapy OR Chemotherapies OR Pharmacotherapy OR Pharmacotherapies):ti,ab,kw

#10 #8 OR #9

#11 MeSH descriptor: [Carcinoma, Non-Small-Cell Lung] explode all trees

#12 Carcinomas, Non-Small-Cell Lung OR Lung Carcinoma, Non-Small-Cell OR Lung Carcinomas, Non-Small-Cell OR Non-Small-Cell Lung Carcinomas OR Non-Small-Cell Lung Carcinoma OR NonSmall Cell Lung Carcinoma OR Carcinoma, Non-Small Cell Lung OR Non-Small Cell Lung Carcinoma OR Non-Small Cell Lung Cancer OR Nonsmall Cell Lung Cancer):ti,ab,kw

#13 #11 OR #12

#14 (Early Stage OR resectable OR operable):ti,ab,kw

#15 #3 AND #7 AND #10 AND #13 AND #14
